# Entropy generation optimization of cilia regulated MHD ternary hybrid Jeffery nanofluid with Arrhenius activation energy and induced magnetic field

**DOI:** 10.1038/s41598-023-41299-8

**Published:** 2023-09-02

**Authors:** Nidhish K. Mishra, Bhupendra K. Sharma, Parikshit Sharma, Taseer Muhammad, Laura M. Pérez

**Affiliations:** 1 Department of Basic Sciences, College of Sciences and Theoretical Studies, Saudi Electronic University, 11673 Riyadh, Saudi Arabia; 2 Department of Mathematics, Birla Institute of Technology and Science, Pilani Campus, Pilani, Rajasthan 333031 India; 3 Department of Mathematics, College of Science, King Khalid University, Abha, 61413 Saudi Arabia; 4 Departamento de Física, FACI, Universidad de Tarapacá, Casilla 7D, 1000000 Arica, Chile

**Keywords:** Mathematics and computing, Applied mathematics, Computational science

## Abstract

This study deals with the entropy generation analysis of synthetic cilia using a ternary hybrid nanofluid (Al–Cu–Fe2O3/Blood) flow through an inclined channel. The objective of the current study is to investigate the effects of entropy generation optimization, heat, and mass transfer on ternary hybrid nanofluid passing through an inclined channel in the proximity of the induced magnetic field. The novelty of the current study is present in studying the combined effect of viscous dissipation, thermophoresis, Brownian motion, exponential heat sink/source, porous medium, endothermic–exothermic chemical reactions, and activation energy in the proximity of induced magnetic field is examined. The governing partial differential equations (PDEs) are transformed into the ordinary differential equations (ODEs) using appropriate transformations. Applying the low Reynolds number and the long-wavelength approximation, resultant ODEs are numerically solved using shooting technique via BVP5C in MATLAB. The velocity, temperature, concentration, and induced magnetism profiles are visually discussed and graphically analyzed for various fluid flow parameters. Graphical analysis of physical interest quantities like mass transfer rate, heat transfer rate, entropy generation optimization, and skin friction coefficient are also graphically discussed. The entropy generation improves for enhancing values of Reynolds number, solutal Grashof number, heat sink/source parameter, Brinkman number, magnetic Prandtl number, and endothermic-exothermic reaction parameter while the reverse effect is noticed for chemical reaction and induced magnetic field parameter. The findings of this study can be applied to enhance heat transfer efficiency in biomedical devices, optimizing cooling systems, designing efficient energy conversion processes, and spanning from renewable energy technologies to aerospace propulsion systems.

## Introduction

Nanofluids are fluid mixtures having nanoparticles of 100 nm or fewer in the base fluid like oil or water. Nanoparticles of metals, oxides, or carbon-based substances have unique characteristics. Argonne National Laboratory scientist Choi^1^ postulated nanofluids in 1995. Alnahdi et al.^2^ investigate the utilization of a ternary Casson hybrid nanofluid in divergent/convergent channels for potential medication applications. This study’s findings have applications in targeted drug delivery systems and thermal therapies. Alnahdi et al.^3^ investigate blood-based ternary hybrid nanofluid flow through a punctured capillary for potential drug delivery applications. The study provides insights into enhancing drug delivery systems using nanofluids, which could optimize therapeutic efficacy. Nanofluids can be further classified based on the number of nanoparticles used to prepare them. Wang et al.’s^4^ investigation into the effects of heat radiation and nanoparticle aggregation on nanofluid flow between a disc and cone gap. The research sheds light on the interplay between aggregation and radiation, which is vital for designing efficient heat transfer systems in many applications. In a ternary hybrid nanofluid flow with a magnetic dipole and nonlinear thermal radiation, Nasir et al.^5^ studied heat transfer. The thermal performance of a ternary hybrid nanofluid flow in an inclined permeable cylinder or plate was investigated by Madhukesh et al.^6^. The findings contribute to the optimization of heat transfer processes involving permeable surfaces and radiative heat exchange. Nanofluids can transmit heat better than basic fluids. Applications of nanofluids can be seen in cooling systems for electronics^7^, industrial operations^8^. Nano fluids’ optical and magnetic properties are useful in energy systems^9^, biomedicine^10^.

Jeffery fluids with their viscoelastic properties have many applications in polymer processing, biomedical engineering, and microfluidics. They enable precise control in processes like extrusion and injection molding, aid in blood flow modeling, and facilitate efficient flow control in lab-on-a-chip devices for chemical analysis and drug delivery. The study has limitations in nanoparticle stability and potential issues with clogging or fouling in the heat exchanger. Using a non-Fourier heat flux model, Sarada et al.^11^ analyse the effects of the exponential form of internal heat generation on water-based ternary hybrid nanofluid flow. The research investigates how fluid dynamics and heat transfer are affected by this heat generating form. The importance of the Stefan blowing effect on the flow and heat transfer of Casson nanofluid through a moving thin needle is explored by Jyothi et al. in their study from Ref.^12^. The paper advances our knowledge of how dynamic conditions influence the behaviour of nanofluids. The electrokinetic peristaltic bioconvective flow of Jeffrey nanofluid with activation energy for binary chemical reaction and changeable fluid characteristics is studied by Hussein et al.^13^. The research of nanofluid flow in biological systems and the examination of variables influencing flow behaviour are the application. Entropy optimisation and heat flux analysis of a Maxwell nanofluid across an exponentially extending surface with velocity slip are the main topics of Nasir et al.’s^14^ study. The study’s accuracy and applicability of the suggested entropy optimisation approach have limits. Li et al.’s^15^ investigation on the dynamics of a mixed Marangoni convective flow with entropy generation involving an aluminium oxide and copper hybrid nanofluid. The study advances knowledge of heat and mass transmission in intricate nanofluid systems.

Cilia are microscopic, hair-like structures that extend from the surface of many types of cells in animals and some plants. In the presence of an angled magnetic field, Munawar et al.^16^ conduct an entropic analysis of the cilia-modulated slip flow of trimetallic nanofluid through an electroosmotic corrugated pump. It has applications in the study of nanofluid flow in microfluidic devices with potential applications in biomedical engineering. Some limitations may include the simplifications and assumptions made in the model, which may not fully capture the complex flow phenomena.

There are many uses for heat sources, including power generation, space heating, water heating, cooking, and industrial activities. They are used in homes, businesses, and industries to provide warmth, hot water, and energy for various thermal applications, contributing to comfort, productivity, and the functioning of various systems. With the aid of hall current and anisotropic slip effects, Umavathi et al.^17^ simulated the swirling hydromagnetic nanofluid flow including gyrotactic microorganisms from a spinning disc to a porous media. The induced magnetic field has numerous applications, including electromagnetic induction, power generation, and electric motors. It is utilized in transformers to transfer electrical energy efficiently, in generators for electricity production, and in electric motors for converting electrical energy into mechanical motion. Nasir et al.’s^18^ investigation highlights the importance of chemical processes and entropy in the Darcy–Forchheimer flow of water and C2H6O2 containing magnetised nanoparticles. This study finds applications in optimizing fluid transport in industrial processes and energy systems. Benos et al.^19^ explore CNT-water nanofluid magnetohydrodynamic natural convection, highlighting the crucial role of nanoparticle aggregations. However, the practical implementations might face challenges in controlling nanoparticle dispersion and aggregation patterns under varying magnetic fields. Benos et al.^20^ analyze unsteady sheet stretching considering slip and magnetohydrodynamic effects.

This research advances the comprehension of heat and mass transfer in stretching processes. Double diffusion convection on peristaltic pumping of Ellis nanofluid because of an induced magnetic field in a non-uniform channel was mathematically calculated by Akram et al.^21^. However, limitations may exist in the assumptions made during the modeling process, which may affect the accuracy of the results. In a hybrid nanofluid flow of Casson material with Hall current through an intricate wavy channel, Li et al.^22^ examine the thermal performance of iron oxide and copper. It has applications in understanding heat transfer enhancement in systems with oscillatory surfaces using hybrid nanofluids. Nasir et al.^23^ investigate the characteristics of MoS2 + GO hybrid nanofluid flow around a whirling sphere, considering nonlinear chemical reactions, radiation, and energy source. This work contributes to understanding fluid behavior in complex systems, with potential applications in thermal management and energy transfer optimization. Punith Gowda et al.’s^24^ analysis of chemically generated non-Newtonian magnetic fluid flow caused by a stretching flat surface.

Thermophoresis and Brownian motion have diverse applications in various fields. Nasir et al.^25^ use the Darcy–Forchheimer model to examine how entropy analysis and radiation affect the movement of MHD advanced nanofluid in a porous media. This study has potential applications in optimizing heat transfer and fluid flow in various porous media systems. Gul et al.^26^ simulate water-based hybrid nanofluids flowing through a porous cavity for heat transfer applications. This research holds promise for enhancing heat exchange processes in porous structures, yet practical implementation might be affected by factors not fully accounted for in the simulation. In order to better understand fluid dynamics in porous medium, Vishalakshi et al.^27^ analyse MHD fluid flow through a porous stretching/shrinking sheet with slips and mass transpiration. This research may be useful for improving procedures involving porous surfaces. The effect of thermophoretic particle deposition on Glauert wall jet slip flow of nanofluid is examined by Alhadhrami et al.^28^. This study sheds light on the particle distribution and heat transfer properties of nanofluid flow under thermophoresis. The effect of Brownian motion and thermophoretic diffusion on the bioconvective micropolar nanofluid flow between double discs with Cattaneo–Christov heat flux is studied by Shahzad et al.^29^. Specific parameter ranges may not have been explored, and the model’s simplifications may not have captured all physical occurrences. Chu et al.’s^30^ investigation of the thermal effects of a hybrid nanofluid caused by a thermo-diffusion-enhanced inclined oscillatory porous surface.

Chemical reactions are integral to numerous applications, including industrial processes, energy production, and materials synthesis. They are crucial for manufacturing fuels, pharmaceuticals, and chemicals, as well as environmental remediation. The effects of chemical reaction, thermal radiation, and heat source on the axisymmetric MHD flow of a jeffrey nanofluid via a ciliated channel filled with a porous media were investigated by Shaheen et al.^31^. The research has implications in biomedical engineering, particularly in understanding fluid flow in biological systems and drug delivery mechanisms. Limitations may arise from the simplifications made in the model, which may not fully capture the complexity of real biological systems. Entropy production in exothermic/endothermic reactive magnetised nanofluid flow across porous curved space with variable permeability and porosity was theoretically analysed by Ullah et al.^32^. Nanofluid flow and heat transmission through a thin moving needle are investigated by Hussain et al.^33^, who consider the role of Arrhenius activation energy and chemical reaction. The phenomena of thermal energy transfer was recently investigated by Sharma et al.^34^, who looked at it in the context of hybrid nanofluid flow caused by a revolving Riga disc. The effects of heat radiation and chemical reaction on this procedure were the primary focus of the study. It’s useful for comprehending how fluid flows and how heat is transferred in magnetohydrodynamic nanofluid systems. For the purpose of performing concurrent endothermic and exothermic processes, Chen^35^ looked into the heat management of thermally coupled reactors. The study has potential applications in industrial processes where heat transfer optimization is crucial for efficient and sustainable operation.

A review of the above literature reveals that the nanofluid flows with the inclusion of various effects has been investigated by many researchers. Though, there is still a gap available in the literature on a cilia regulated ternary hybrid Jeffery nanofluid through a peristaltic channel with the consideration of various effects. The present article fills the gap with the consideration of ternary hybrid nano molecules on the fluid through a cilia regulated peristaltic channel with induced magnetic effect which never investigated before. Optimising entropy production, heat, and mass transfer in a ternary hybrid nanofluid as it flows down an inclined channel in the presence of an induced magnetic field and a heat source is also a key focus of this research. Cilia play important roles in human tissues and vital organs, such as cell migration and external fluid movement, therefore this study may benefit the medical field. The considered slanted geometry also gives crucial measurements for endoscopes and microscopes. For this reason, this investigation attempts to answer the following questions:

1. What are the significant effects of ternary hybrid nanoparticles controlled by the cilia on the Sherwood number, Nusselt number, temperature, and skin friction coefficient?
2. What impact do Brinkman number, magnetic Prandtl number, endothermic-exothermic reaction parameter have on optimization of entropy generation?
3. How do the endothermic-exothermic chemical reactions influence the condensed and restrained skin friction factor, Nusselt number, and Sherwood number?
4. How the induced magnetic field affects the dynamics of a fluid with mixed convection hybrid nanofluid through inclined channel?
5. How do skin friction, Bejan number, and entropy formation physically relate to exponential heat source/sink and activation energy? How fast do heat and mass transfer rates change?

## Mathematical formulation

**Figure 1:**
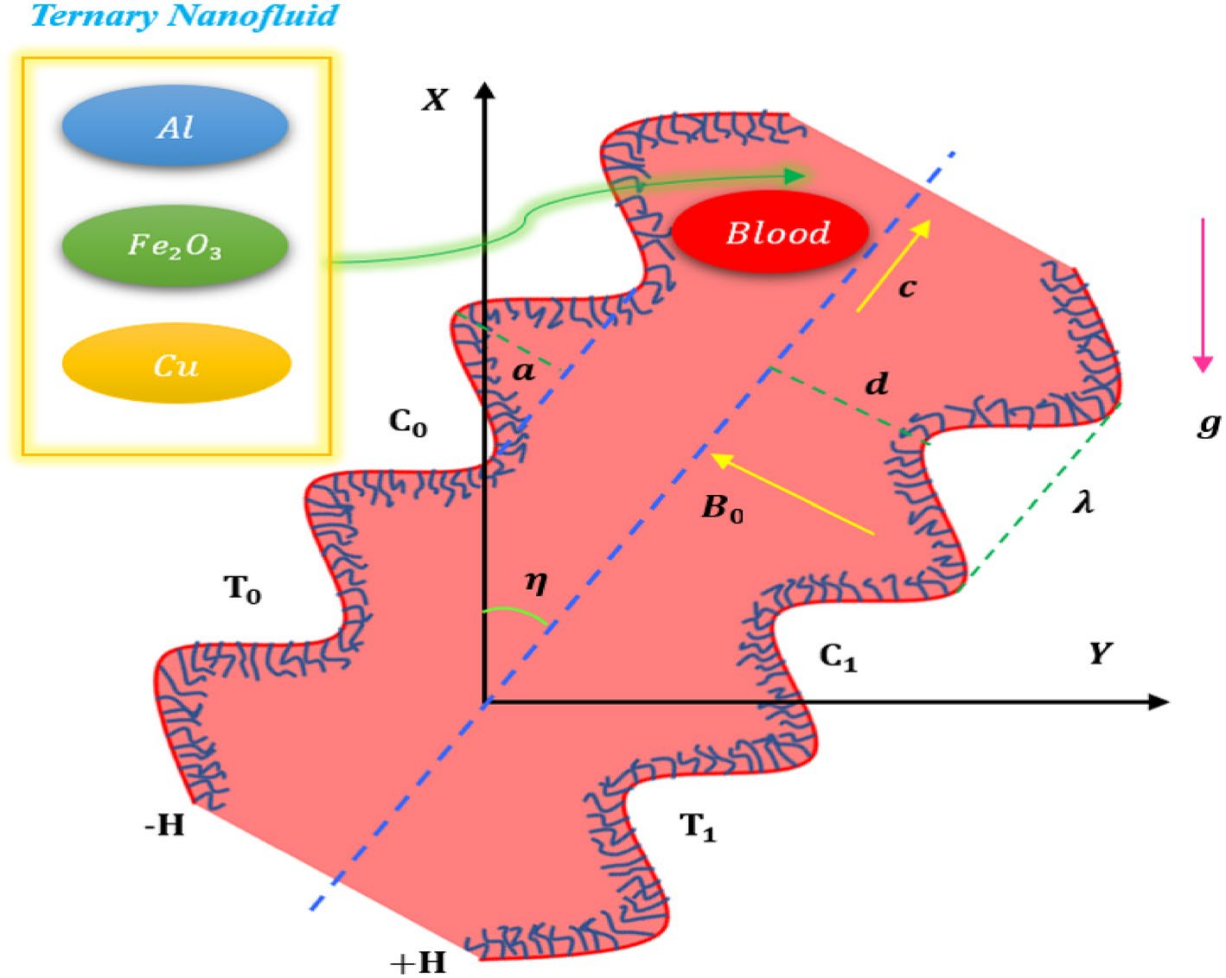
Geometrical description of the cilia problem.

Considered a laminar viscous, incompressible thermally and electrically conducting ${\text{Al}}{-}{\text{Cu}}{-}{{\text{Fe}}_{2}}{{\text{O}}_{3}}{-}Blood$ ternary hybrid Jeffery nanofluid passing through an infinite two-dimensional inclined channel of thickness 2d. Cartesian coordinate system is considered in which the X-axis is oriented in the vertical direction, and Y-axis is oriented in the horizontal direction. Peristaltic flow is induced by traveling sinusoidal waves advancing with constant velocity c. Assume that the inner walls of the tube are covered with an unlimited number of constantly beating cilia, which will result in a symplectic metachronal wave that travels along the positive x-axis with an inclination angle $\eta$ and a wave speed of c, as illustrated in Fig. 1. The medium between the cilia walls is porous having ${k}_{1}$ as the permeability constant. The flow is given a homogeneous magnetic field from outside. The magnetic field has a strength of ${B}_{0}$ and acts perpendicular to the flow direction. The channel is inclined at an angle of $\eta$ with the vertical axis. Exponential heat source is considered with ${q}_{0}<0$ heat sink and ${q}_{0}>0$ heat source. Endothermic and exothermic chemical reactions with activation energy are considered in heat energy and concentration equations as per the Arrhenius law. The Cauchy stress tensor T, extra stress tensor S, for an incompressible Jeffrey fluid, are defined as^36^:

$$T=-PI+S,$$

$$S=\frac{{\mu }_{f}}{1+{\lambda }_{1}}\left(\dot{\gamma }+{\lambda }_{2}^{*}\frac{d\dot{\gamma }}{d{t}^{*}}\right),$$

where, P is the pressure, and I is the identity tensor.

The upper and lower ciliated walls are mathematically governed by Ref.^37^:

1 $$+H=d+\left({X}^{*}-c{t}^{*}\right)G+L\left\{{a}_{1}\mathrm{sin}\left[\frac{{b}_{1}}{\lambda }\left({X}^{*}-c{t}^{*}\right)\right]+{a}_{2}\mathrm{sin}\left[\frac{{b}_{2}}{\lambda }\left({X}^{*}-c{t}^{*}\right)\right]+{a}_{3}\mathrm{sin}\left[\frac{{b}_{3}}{\lambda }\left({X}^{*}-c{t}^{*}\right)\right]\right\},$$

2 $$-H=-d-\left({X}^{*}-c{t}^{*}\right)G-L\left\{{a}_{1}\mathrm{sin}\left[\frac{{b}_{1}}{\lambda }\left({X}^{*}-c{t}^{*}\right)\right]+{a}_{2}\mathrm{sin}\left[\frac{{b}_{2}}{\lambda }\left({X}^{*}-c{t}^{*}\right)\right]+{a}_{3}\mathrm{sin}\left[\frac{{b}_{3}}{\lambda }\left({X}^{*}-c{t}^{*}\right)\right]\right\}.$$

The non-uniform parameter G < 0 represents the convergent regime, and G > 0 represents the divergent regime. The prerequisite for both cilia walls is described as follows: $d < {a}_{1}+{a}_{2}+{a}_{3}$. With the aforementioned presumptions, the governing equations for this fluid can be expressed as follows^36–43^.

Continuity equation:

3 $$\frac{\partial {U}^{*}}{\partial {X}^{*}}+\frac{\partial {W}^{*}}{\partial {Y}^{*}}=0.$$

Solenoidal equation:

4 $$\frac{\partial {m}_{x}^{*}}{\partial {X}^{*}}+\frac{\partial {m}_{y}^{*}}{\partial {Y}^{*}}=0.$$

Momentum equations:

5 $$\begin{aligned} & {\rho }_{thnf}\left[\frac{\partial {U}^{*}}{\partial {t}^{*}}+{U}^{*}\frac{\partial {U}^{*}}{\partial {X}^{*}}+{W}^{*}\frac{\partial {U}^{*}}{\partial {Y}^{*}}\right]=-\frac{\partial {P}^{*}}{\partial {X}^{*}}+\frac{\partial {S}_{{X}^{*}{X}^{*}}^{*}}{\partial {X}^{*}}+\frac{\partial {S}_{{X}^{*}{Y}^{*}}^{*}}{\partial {Y}^{*}}+{\rho }_{thnf}g{\beta }_{t}\left(1-{C}_{0}\right)\left({T}^{*}-{T}_{0}\right)\mathrm{cos}\eta \\ &\quad-\left({\rho }_{p}-{\rho }_{f}\right)g{\beta }_{c}\left({C}^{*}-{C}_{0}\right)\mathrm{cos}\eta -{\sigma }_{thnf}{B}_{0}^{2}{U}^{*}-\frac{{\mu }_{thnf}}{{k}_{1}}{U}^{*}-{F}_{0}{U}^{*2}+{\mu }_{m}\left({m}_{y}^{*}\frac{\partial {m}_{x}^{*}}{\partial {X}^{*}}+{m}_{x}^{*}\frac{\partial {m}_{x}^{*}}{\partial {Y}^{*}}\right),\end{aligned}$$

6 $${\rho }_{thnf}\left[\frac{\partial {W}^{*}}{\partial {t}^{*}}+{U}^{*}\frac{\partial {W}^{*}}{\partial {X}^{*}}+{W}^{*}\frac{\partial {W}^{*}}{\partial {Y}^{*}}\right]=-\frac{\partial {P}^{*}}{\partial {Y}^{*}}+\frac{\partial {S}_{{X}^{*}{Y}^{*}}^{*}}{\partial {X}^{*}}+\frac{\partial {S}_{{Y}^{*}{Y}^{*}}^{*}}{\partial {Y}^{*}}-\frac{{\mu }_{thnf}}{{k}_{1}}{W}^{*}-{F}_{0}{W}^{*2}+{\mu }_{m}\left({m}_{y}^{*}\frac{\partial {m}_{y}^{*}}{\partial {X}^{*}}+{m}_{x}^{*}\frac{\partial {m}_{y}^{*}}{\partial {Y}^{*}}\right).$$

Energy equation:

7 $$\begin{aligned} & {\left(\rho {c}_{p}\right)}_{thnf}\left[\frac{\partial {T}^{*}}{\partial {t}^{*}}+{U}^{*}\frac{\partial {T}^{*}}{\partial {X}^{*}}+{W}^{*}\frac{\partial {T}^{*}}{\partial {Y}^{*}}\right]={k}_{thnf}\left(\frac{{\partial }^{2}{T}^{*}}{\partial {X}^{*2}}+\frac{{\partial }^{2}{T}^{*}}{\partial {Y}^{*2}}\right)\\ &\quad+\left[{S}_{{X}^{*}{X}^{*}}^{*}\frac{\partial {U}^{*}}{\partial {X}^{*}}+{S}_{{X}^{*}{Y}^{*}}^{*}\left(\frac{\partial {U}^{*}}{\partial {Y}^{*}}+\frac{\partial {W}^{*}}{\partial {X}^{*}}\right)+{S}_{{Y}^{*}{Y}^{*}}^{*}\frac{\partial {W}^{*}}{\partial {X}^{*}}\right]\\ &\quad+{\tau \left(\rho {c}_{p}\right)}_{thnf}\left[{D}_{B}\left(\frac{\partial {T}^{*}}{\partial {X}^{*}}\frac{\partial {C}^{*}}{\partial {X}^{*}}+\frac{\partial {T}^{*}}{\partial {Y}^{*}}\frac{\partial {C}^{*}}{\partial {Y}^{*}}\right)+\frac{{D}_{T}}{{T}_{0}}\left\{{\left(\frac{\partial {T}^{*}}{\partial {X}^{*}}\right)}^{2}+{\left(\frac{\partial {T}^{*}}{\partial {Y}^{*}}\right)}^{2}\right\}\right]\\ &\quad+{q}_{o}\left({T}^{*}-{T}_{0}\right){e}^{\frac{{-Y}^{*}}{d}}+{k}_{r}^{2}\varsigma \left({C}^{*}-{C}_{0}\right){R}_{c}.\end{aligned}$$

Concentration equation:

8 $$\frac{\partial {C}^{*}}{\partial {t}^{*}}+{U}^{*}\frac{\partial {C}^{*}}{\partial {X}^{*}}+{W}^{*}\frac{\partial {C}^{*}}{\partial {Y}^{*}}={D}_{B}\left(\frac{{\partial }^{2}{C}^{*}}{\partial {X}^{*2}}+\frac{{\partial }^{2}{C}^{*}}{\partial {Y}^{*2}}\right)+\frac{{D}_{T}}{{T}_{0}}\left(\frac{{\partial }^{2}{T}^{*}}{\partial {X}^{*2}}+\frac{{\partial }^{2}{T}^{*}}{\partial {Y}^{*2}}\right)-{k}_{r}^{2}\left({C}^{*}-{C}_{0}\right){R}_{c}.$$

Induced magnetic field equation:

9 $$\frac{\partial {m}_{x}^{*}}{\partial {t}^{*}}+{U}^{*}\frac{\partial {m}_{x}^{*}}{\partial {X}^{*}}+{W}^{*}\frac{\partial {m}_{x}^{*}}{\partial {Y}^{*}}={m}_{y}^{*}\frac{\partial {U}^{*}}{\partial {X}^{*}}+{m}_{x}^{*}\frac{\partial {U}^{*}}{\partial {Y}^{*}}+{\alpha }_{m}\frac{{\partial }^{2}{m}_{x}^{*}}{\partial {Y}^{*2}},$$

where ${\rho }_{thnf}, {\sigma }_{thnf}, {\mu }_{thnf}, {k}_{thnf}$ and ${\left({C}_{p}\right)}_{thnf}$ are the ternary hybrid nanofluid’s density, electrical conductivity, dynamic viscosity, thermal conductivity, and specific heat, in that order. The values of these thermophysical parameters for the existing ternary hybrid nanofluid are listed in Table 1 and are mathematically expressed as^3^:

**Table 1:** Thermophysical properties of ternary hybrid nanofluid.

Properties	Notation	Nanoparticles			Base fluid
		Al	Cu	Fe_2_O_3_	Blood
Specific heat (J/kg K)	${C}_{p}$	903	385	650	3617
Density (kg/m^3^)	$\rho$	2710	8933	5240	1150
Thermal conductivity (W/m K)	$k$	205	400	20	0.53

10 $$\left.\begin{array}{c}\frac{{\mu }_{thnf}}{{\mu }_{f}}={\left[\left(1-{\phi }_{Al}\right)\left(1-{\phi }_{F{e}_{2}{O}_{3}}\right)\left(1-{\phi }_{Cu}\right)\right]}^{-2.5},\\ \frac{{\rho }_{thnf}}{{\rho }_{f}}=\left(1-{\phi }_{Al}\right)\left[\left(1-{\phi }_{F{e}_{2}{O}_{3}}\right)\left\{\left(1-{\phi }_{Cu}\right)+{\phi }_{Cu}\frac{{\rho }_{Cu}}{{\rho }_{f}}\right\}+{\phi }_{F{e}_{2}{O}_{3}}\frac{{\rho }_{F{e}_{2}{O}_{3}}}{{\rho }_{f}}\right]+{\phi }_{Al}\frac{{\rho }_{Al}}{{\rho }_{f}},\\ \frac{{(\rho {C}_{p})}_{thnf}}{{(\rho {C}_{p})}_{f}}=\left(1-{\phi }_{Al}\right)\left[\left(1-{\phi }_{F{e}_{2}{O}_{3}}\right)\left\{\left(1-{\phi }_{Cu}\right)+{\phi }_{Cu}\frac{{(\rho {C}_{p})}_{Cu}}{{(\rho {C}_{p})}_{f}}\right\}+{\phi }_{F{e}_{2}{O}_{3}}\frac{{(\rho {C}_{p})}_{F{e}_{2}{O}_{3}}}{{(\rho {C}_{p})}_{f}}\right]+{\phi }_{Al}\frac{{(\rho {C}_{p})}_{Al}}{{(\rho {C}_{p})}_{f}},\\ \frac{{\sigma }_{thnf}}{{\sigma }_{hnf}}=\left[\frac{{\sigma }_{Cu}+2{\sigma }_{hnf}-2{\phi }_{Cu}\left({\sigma }_{hnf}-{\sigma }_{Cu}\right)}{{\sigma }_{Cu}+2{\sigma }_{hnf}+{\phi }_{Cu}\left({\sigma }_{hnf}-{\sigma }_{Cu}\right)}\right],\\ \frac{{\sigma }_{hnf}}{{\sigma }_{nf}}=\left[\frac{{\sigma }_{F{e}_{2}{O}_{3}}+2{\sigma }_{nf}-2{\phi }_{F{e}_{2}{O}_{3}}\left({\sigma }_{nf}-{\sigma }_{F{e}_{2}{O}_{3}}\right)}{{\sigma }_{F{e}_{2}{O}_{3}}+2{\sigma }_{nf}+{\phi }_{F{e}_{2}{O}_{3}}\left({\sigma }_{nf}-{\sigma }_{F{e}_{2}{O}_{3}}\right)}\right],\\ \frac{{\sigma }_{nf}}{{\sigma }_{f}}=\left[\frac{{\sigma }_{Al}+2{\sigma }_{f}-2{\phi }_{Al}\left({\sigma }_{f}-{\sigma }_{Al}\right)}{{\sigma }_{Al}+2{\sigma }_{f}+{\phi }_{Al}\left({\sigma }_{f}-{\sigma }_{Al}\right)}\right],\\ \frac{{k}_{thnf}}{{k}_{hnf}}=\left[\frac{{k}_{Cu}+2{k}_{Cu}-2{\phi }_{Cu}\left({k}_{hnf}-{k}_{Cu}\right)}{{k}_{Cu}+2{k}_{Cu}+{\phi }_{Cu}\left({k}_{hnf}-{k}_{Cu}\right)}\right],\\ \frac{{k}_{hnf}}{{k}_{nf}}=\left[\frac{{k}_{F{e}_{2}{O}_{3}}+2{k}_{nf}-2{\phi }_{F{e}_{2}{O}_{3}}\left({k}_{nf}-{k}_{F{e}_{2}{O}_{3}}\right)}{{k}_{F{e}_{2}{O}_{3}}+2{k}_{nf}+{\phi }_{F{e}_{2}{O}_{3}}\left({k}_{nf}-{k}_{F{e}_{2}{O}_{3}}\right)}\right],\\ \frac{{k}_{nf}}{{k}_{f}}=\left[\frac{{k}_{Al}+2{k}_{f}-2{\phi }_{Al}\left({k}_{f}-{k}_{Al}\right)}{{k}_{Al}+2{k}_{f}+{\phi }_{Al}\left({k}_{f}-{k}_{Al}\right)}\right],\\ \phi ={\phi }_{Cu}+{\phi }_{F{e}_{2}{O}_{3}}+{\phi }_{Al}\end{array}\right\}.$$

The following transformations are used to convert the considered fluid flow problem from fixed frame to wave frame:

11 $${X}^{*}={x}^{*}-c{t}^{*}, {Y}^{*}={y}^{*}, {U}^{*}={u}^{*}+c,{W}^{*}={w}^{*}, {P}^{*}\left({X}^{*},{Y}^{*},{t}^{*}\right)=p\left({x}^{*},{y}^{*}\right), {T}^{*}=T, {C}^{*}=C.$$

Introducing the following dimensional parameters:

$$x=\frac{{x}^{*}}{\lambda }, y=\frac{{y}^{*}}{d}, u=\frac{{u}^{*}}{c}, w=\frac{{w}^{*}}{c}, t=\frac{c{t}^{*}}{\lambda }, h=\frac{H}{d}, \delta =\frac{d}{\lambda },p=\frac{{d}^{2}{p}^{*}}{c\lambda {\mu }_{f}}, {\lambda }_{2}^{*}=\frac{c{\lambda }_{2}}{d}, S=\frac{{S}^{*}d}{c{\mu }_{f}},{r}_{i}=\frac{{a}_{i}}{d},$$

12 $${q}_{i}=\frac{{b}_{i}}{\lambda },{m}_{x}^{*}=\frac{{m}_{0}{m}_{x}}{\sqrt{Re}}, {m}_{y}^{*}=\frac{{m}_{0}{m}_{y}}{\sqrt{Re}}, \psi =\frac{{\psi }^{*}}{cd}, \theta =\frac{{T}^{*}-{T}_{0}}{{T}_{1}-{T}_{0}},\Theta =\frac{{C}^{*}-{C}_{0}}{{C}_{1}-{C}_{0}},\Omega =\frac{{T}_{1}-{T}_{0}}{{T}_{0}},\Pi =\frac{{{\mathrm{C}}_{1}-\mathrm{C}}_{0}}{{C}_{0}},$$

where, $p$ is pressure, $\theta$ is dimensionless temperature, $\Theta$ is dimensionless concentration, $\Omega$ is the temperature ratio, and $\Pi$ is concentration ratio. The rate constant of endothermic and exothermic chemical reactions with activation energy, ${R}_{c}$ is assumed to be dependent on the absolute temperature ${T}^{*}$ and is provided by the modified Arrhenius law^44,45^:

$${R}_{c}={\left(\frac{{T}^{*}}{{T}_{0}}\right)}^{n}{e}^{-\left(\frac{{E}_{a}}{{k}_{b}{T}^{*}}\right)}.$$

Thus, Eqs. (5, 6, 7, 8, 9) simplify to the dimensionless form as follows:

13 $$\begin{aligned} & {A}_{1}Re\delta \left[\left(1+u\right)\frac{\partial u}{\partial x}+\frac{w}{\delta }\frac{\partial u}{\partial y}\right]=-\frac{\partial p}{\partial x}+\delta \frac{\partial {S}_{xx}}{\partial x}+\frac{\partial {S}_{xy}}{\partial y}+{A}_{1}Gr\theta \mathrm{cos}\eta -Gc\mathrm{\Theta cos}\eta\\ &\quad -\left[{A}_{3}{M}^{2}+\frac{{A}_{2}}{Da}+ReFr\left(1+u\right)\right]\left(1+u\right)+{M}_{f}\left(\delta {m}_{y}\frac{\partial {m}_{x}}{\partial x}+{m}_{x}\frac{\partial {m}_{x}}{\partial y}\right),\end{aligned}$$

14 $${A}_{1}Re{\delta }^{2}\left[\left(1+u\right)\frac{\partial w}{\partial x}+\frac{w}{\delta }\frac{\partial w}{\partial y}\right]=-\frac{\partial p}{\partial y}+{\delta }^{2}\frac{\partial {S}_{xy}}{\partial x}+\delta \frac{\partial {S}_{yy}}{\partial y}+\delta {M}_{f}\left(\delta {m}_{y}\frac{\partial {m}_{x}}{\partial x}+{m}_{x}\frac{\partial {m}_{x}}{\partial y}\right)-{A}_{2}\delta \frac{w}{Da}-\delta ReFr{w}^{2},$$

15 $$\begin{aligned} & {A}_{4}Re\delta \left[\left(1+u\right)\frac{\partial \theta }{\partial x}+\frac{w}{\delta }\frac{\partial \theta }{\partial y}\right]=\frac{{A}_{5}}{Pr}\left({\delta }^{2}\frac{{\partial }^{2}\theta }{\partial {x}^{2}}+\frac{{\partial }^{2}\theta }{\partial {y}^{2}}\right)+Ec\left[+\delta {S}_{xx}\frac{\partial u}{\partial x}+{S}_{xx}\left(\frac{\partial u}{\partial y}+\delta \frac{\partial w}{\partial x}\right)+{\delta S}_{yy}\frac{\partial w}{\partial y}\right]\\ &\quad+Nb\left({\delta }^{2}\frac{\partial \theta }{\partial x}\frac{\partial\Theta }{\partial x}+\frac{\partial \theta }{\partial y}\frac{\partial\Theta }{\partial y}\right)+Nt\left[{\delta }^{2}{\left(\frac{\partial \theta }{\partial x}\right)}^{2}+{\left(\frac{\partial \theta }{\partial y}\right)}^{2}\right]+Q\theta {e}^{-y}+K{r}_{1}K{r}_{2}\Theta {\left(1+\Omega \theta \right)}^{n}{e}^{-\left(\frac{{E}^{*}}{1+\Omega \theta }\right)},\end{aligned}$$

16 $$ScRe\delta \left[\left(1+u\right)\frac{\partial\Theta }{\partial x}+\frac{w}{\delta }\frac{\partial\Theta }{\partial y}\right]=\left({\delta }^{2}\frac{{\partial }^{2}\Theta }{\partial {x}^{2}}+\frac{{\partial }^{2}\Theta }{\partial {y}^{2}}\right)+\frac{Nt}{Nb}\left({\delta }^{2}\frac{{\partial }^{2}\theta }{\partial {x}^{2}}+\frac{{\partial }^{2}\theta }{\partial {y}^{2}}\right)-K{r}_{1}Sc\Theta {\left(1+\Omega \theta \right)}^{n}{e}^{-\left(\frac{{E}^{*}}{1+\Omega \theta }\right)},$$

17 $${Pr}_{m}Re\delta \left[\left(1+u\right)\frac{\partial {m}_{x}}{\partial x}+\frac{w}{\delta }\frac{\partial {m}_{x}}{\partial y}\right]=\frac{{\partial }^{2}{m}_{x}}{\partial {y}^{2}}+{Pr}_{m}Re\left(\delta {m}_{y}\frac{\partial u}{\partial x}+{m}_{x}\frac{\partial u}{\partial y}\right),$$

where ${A}_{1-4}$ represent the ratios of ternary hybrid nanofluid thermophysical properties to that of base fluid and defined as follows:

$${A}_{1}=\frac{{\rho }_{thnf}}{{\rho }_{f}}, {A}_{2}=\frac{{\mu }_{thnf}}{{\mu }_{f}}, {A}_{3}=\frac{{\sigma }_{thnf}}{{\sigma }_{f}}, {A}_{4}=\frac{{\left(\rho {c}_{p}\right)}_{thnf}}{{\left(\rho {c}_{p}\right)}_{f}}, {A}_{5}=\frac{{k}_{thnf}}{{k}_{f}}.$$

The non-dimensional parameters used in Eqs. (13, 14, 15, 16, 17) are described as follows:

$Gr$ (Thermal Grashof number) $=\frac{{\rho }_{f}g{\beta }_{t}\left(1-{C}_{0}\right)\left(T-{T}_{0}\right){d}^{2}}{{\mu }_{f}c}$, $Gc$ (Solutal Grashof number) $=\frac{\left({\rho }_{p}-{\rho }_{f}\right)g{\beta }_{c}\left({C}^{*}-{C}_{0}\right){d}^{2}}{{\mu }_{f}c}$, $Re$ (Reynolds number) $=\frac{cd}{{v}_{f}}$, $M$ (Magnetic number) $=\sqrt{\frac{{\sigma }_{f}}{{\mu }_{f}}}{B}_{0}d$, $Da$ (Darcy number) $=\frac{{k}_{1}}{{d}^{2}}$, $Fr$ (Forchheimer number)$=\frac{{F}_{0}d}{{\rho }_{f}}$, $Ec$ (Eckert number) $=\frac{{c}^{2}}{{\left({c}_{p}\right)}_{f}\left({T}_{1}-{T}_{0}\right)}$, $Pr$ (Prandtl number) $=\frac{{\mu }_{f}{\left({c}_{p}\right)}_{f}}{{k}_{f}}$, $Nb$ (Brownian motion parameter) $=\frac{\tau {D}_{B}{(C}_{1}-{C}_{0})}{{v}_{f}}$, $Nt$ (Thermophoresis parameter) $=\frac{\tau {D}_{T}\left({T}_{1}-{T}_{0}\right)}{{T}_{0}{v}_{f}}$, $K{r}_{1}$ (Chemical reaction parameter) $=\frac{{k}_{r}^{2}{d}^{2}}{{v}_{f}}$, $K{r}_{2}$ (Endothermic-exothermic reaction parameter) $=\frac{\varsigma }{{\left(\rho {c}_{p}\right)}_{f}}\left(\frac{{C}_{1}-{C}_{0}}{{T}_{1}-{T}_{0}}\right)$, ${E}^{*}$ (Activation energy parameter)$=\frac{{E}_{a}}{{K}_{b}{T}_{0}}$, $Br$ (Brinkman number) $=PrEc$, $Le$ (Lewis number) $=\frac{{k}_{f}}{{D}_{B}{C}_{0}}$, $Sc$ (Schmidt number) $=\frac{{v}_{f}}{{D}_{B}}$, $P{r}_{m}$ (Magnetic Prandtl number) $=\frac{{V}_{f}}{{\alpha }_{m}}$, ${M}_{f}$ (Induced magnetic field number) $=\frac{{\mu }_{m}{m}_{0}^{2}}{{\rho }_{f}^{2}{c}^{2}}$, $Q$ (Heat generation parameter) $=\frac{{q}_{o}{d}^{2}}{{\left(\rho {c}_{p}\right)}_{f}{v}_{f}}.$

Introducing the stream function $\psi$ and $\varphi$ for velocity fields and induced magnetic fields, respectively:

$$u=\frac{\partial \psi }{\partial y}, w=-\delta \frac{\partial \psi }{\partial x}, {m}_{x}=\frac{\partial \varphi }{\partial y}, {m}_{y}=\frac{\partial \varphi }{\partial x}.$$

Hence, the following form represent the stress components:

$${S}_{xx}=\frac{2\delta }{1+{\lambda }_{1}}\left[1+\frac{{\lambda }_{2}c\delta }{d}\left(\frac{\partial \psi }{\partial y}\frac{\partial }{\partial x}-\frac{\partial \psi }{\partial x}\frac{\partial }{\partial y}\right)\right]\frac{\partial }{\partial y}\left(\frac{\partial \psi }{\partial x}\right),$$

$${S}_{xy}=\frac{1}{1+{\lambda }_{1}}\left[1+\frac{{\lambda }_{2}c\delta }{d}\left(\frac{\partial \psi }{\partial y}\frac{\partial }{\partial x}-\frac{\partial \psi }{\partial x}\frac{\partial }{\partial y}\right)\right]\left(\frac{{\partial }^{2}\psi }{\partial {y}^{2}}-\delta \frac{{\partial }^{2}\psi }{\partial {x}^{2}}\right),$$

$${S}_{yy}=\frac{2\delta }{1+{\lambda }_{1}}\left[1+\frac{{\lambda }_{2}c\delta }{d}\left(\frac{\partial \psi }{\partial y}\frac{\partial }{\partial x}-\frac{\partial \psi }{\partial x}\frac{\partial }{\partial y}\right)\right]\frac{\partial }{\partial y}\left(\frac{\partial \psi }{\partial x}\right).$$

By using the long wavelength ($\lambda \to \infty \Rightarrow \delta \to 0)$ and low Reynolds number $(Re\to 0)$ approximations in the above-mentioned stress components and dimensionless governing Eqs. (13, 14, 15, 16, 17) become:

18 $$\frac{\partial p}{\partial x}=\left({A}_{2}+\frac{1}{1+{\lambda }_{1}}\right)\frac{{\partial }^{3}\psi }{\partial {y}^{3}}+{A}_{1}Gr\theta \mathrm{cos}\eta -Gc\mathrm{\Theta cos}\eta -\left[{A}_{3}{M}^{2}+\frac{{A}_{2}}{Da}+ReFr\left(1+\frac{\partial \psi }{\partial y} \right)\right]\left(1+\frac{\partial \psi }{\partial y} \right)+{M}_{f}\frac{\partial \varphi }{\partial y}\frac{{\partial }^{2}\varphi }{\partial {y}^{2}},$$

19 $$\frac{\partial p}{\partial y}=0,$$

20 $${A}_{5}\frac{{\partial }^{2}\theta }{\partial {y}^{2}}+Br\left[{A}_{2}{\left(\frac{{\partial }^{2}\psi }{\partial {y}^{2}}\right)}^{2}\right]+PrNb\frac{\partial \theta }{\partial y}\frac{\partial\Theta }{\partial y}+PrNt{\left(\frac{\partial \theta }{\partial y}\right)}^{2}+Q\theta {e}^{-y}+PrK{r}_{1}K{r}_{2}\Theta {\left(1+\Omega \theta \right)}^{n}{e}^{-\left(\frac{{E}^{*}}{1+\Omega \theta }\right)}=0,$$

21 $$\frac{{\partial }^{2}\Theta }{\partial {y}^{2}}+\frac{Nt}{Nb}\frac{{\partial }^{2}\theta }{\partial {y}^{2}}-K{r}_{1}Sc\Theta {\left(1+\Omega \theta \right)}^{n}{e}^{-\left(\frac{{E}^{*}}{1+\Omega \theta }\right)}=0,$$

22 $$\frac{{\partial }^{3}\varphi }{\partial {y}^{3}}=-{Pr}_{m}Re\frac{\partial \varphi }{\partial y}\frac{{\partial }^{2}\psi }{\partial {y}^{2}}.$$

From Eq. (18), the pressure gradient is eliminated via cross-difference. The resulting equation is obtained as follows:

23 $$\begin{aligned} & \left({A}_{2}+\frac{1}{1+{\lambda }_{1}}\right)\frac{{\partial }^{4}\psi }{\partial {y}^{4}}-\left[{A}_{3}{M}^{2}+\frac{{A}_{2}}{Da}+2ReFr\left(1+\frac{\partial \psi }{\partial y} \right)\right]\frac{{\partial }^{2}\psi }{\partial {y}^{2}}\\ &\quad+{A}_{1}Gr\frac{\partial \theta }{\partial y}\mathrm{cos}\eta -Gc\frac{\partial\Theta }{\partial y}\mathrm{cos}\eta +{M}_{f}\left[{\left(\frac{{\partial }^{2}\varphi }{\partial {y}^{2}}\right)}^{2}+\frac{\partial \varphi }{\partial y}\frac{{\partial }^{3}\varphi }{\partial {y}^{3}}\right]=0.\end{aligned}$$

With the following boundary conditions^46^:

$$\psi =+\frac{F}{2}, \frac{\partial \psi }{\partial y}=\frac{1}{1-L\left[{r}_{1}{q}_{1}\mathrm{cos}\left({q}_{1}x\right)+{r}_{2}{q}_{2}\mathrm{cos}\left({q}_{2}x\right)+{r}_{3}{q}_{3}\mathrm{cos}\left({q}_{3}x\right)\right]},\theta =0,\Theta =0, \chi =0, \frac{{\partial }^{2}\varphi }{\partial {y}^{2} }=0\,at\,y=-h,$$

24 $$\psi =-\frac{F}{2}, \frac{\partial \psi }{\partial y}=\frac{1}{1-L\left[{r}_{1}{q}_{1}\mathrm{cos}\left({q}_{1}x\right)+{r}_{2}{q}_{2}\mathrm{cos}\left({q}_{2}x\right)+{r}_{3}{q}_{3}\mathrm{cos}\left({q}_{3}x\right)\right]},\theta =1,\Theta =1, \chi =1, \frac{\partial \varphi }{\partial y}=1\,at\,y=+h,$$

### Engineering quantities of interests

This study also analyses the relevant physical parameters including shear stress, mass transfer rate, and heat transfer rate. The mathematical formulas for the Nusselt number, skin friction coefficient, and Sherwood number are^47,48^:

25 $$Nu=\frac{d{q}_{w}}{{k}_{f}\left({T}_{1}-{T}_{0}\right)}, {C}_{f}=\frac{{\tau }_{u}}{{\rho }_{f}{c}^{2}}, Sh=\frac{d{m}_{w}}{{\rho }_{f}{D}_{B}({C}_{1}-{C}_{0})},$$

where, ${q}_{w}$ is the heat flux, ${\tau }_{u}$ is the axial stress, and ${m}_{w}$ is the mass flux. They are defined as:

26 $${q}_{w}=-{k}_{thnf}{\left( \frac{\partial {T}^{*}}{\partial {Y}^{*}}\right)}_{{Y}^{*}=0}, {\tau }_{u}={\mu }_{thnf}{\left( \frac{\partial {U}^{*}}{\partial {Y}^{*}}\right)}_{{Y}^{*}=0}, {m}_{w}= -{\rho }_{f}{D}_{B}{\left( \frac{\partial {C}^{*}}{\partial {Y}^{*}}\right)}_{{Y}^{*}=0}.$$

The above expressions’ non-dimensional forms are as follows:

27 $$Nu=-{A}_{5}{\theta }{^\prime}\left(0\right), {C}_{f}Re ={A}_{2}{u}{^\prime}\left(0\right), Sh=-{\Theta }{^\prime}\left(0\right).$$

### Entropy generation

Now, we will discuss the entropy generation analysis for the considered problem. It is noted that the impacts of heat transfer, mass transfer, thermal radiation, fluid friction, Joule heating, homogeneous and heterogeneous chemical reaction are the main causes of entropy in the medium. Considering the second rule of thermodynamics, the following is the expression for entropy generation^49^:

28 $$\begin{aligned} {S}_{gen}&=\frac{{k}_{thnf}}{{T}_{0}^{2}}{\left(\nabla {T}^{*}\right)}^{2}+\frac{{\mu }_{thnf}}{{T}_{0}}{\left(\frac{{\partial }^{2}{\psi }^{*}}{\partial {y}^{*2}}\right)}^{2}+\frac{{\mu }_{thnf}}{{T}_{0}}\left(\frac{1}{1+{\lambda }_{1}}\right){\left(\frac{{\partial }^{2}{\psi }^{*}}{\partial {y}^{*2}}\right)}^{2}\\ &\quad+\frac{{\sigma }_{thnf}{B}_{0}^{2}}{{T}_{0}}{\left(c+\frac{\partial {\psi }^{*}}{\partial {y}^{*}}\right)}^{2}+\frac{{D}_{B}}{{C}_{0}}{\left(\nabla {C}^{*}\right)}^{2}+\frac{{D}_{B}}{{T}_{0}}\left(\nabla {T}^{*}\nabla {C}^{*}\right).\end{aligned}$$

Using Eqs. (11) and (12) in Eq. (28), the characteristic entropy, lubrication approximations, and the formula for the total entropy generation number yield the following:

29 $$N_{G} = \underbrace {{A_{5} \left( {\frac{\partial \theta }{{\partial y}}} \right)^{2} }}_{{N_{T} }} + \underbrace {{\frac{Br}{{\Omega }}\left( {\frac{{\partial^{2} \psi }}{{\partial y^{2} }}} \right)^{2} }}_{{N_{V} }} + \underbrace {{A_{2} \frac{Br}{{\Omega }}\left( {\frac{1}{{1 + \lambda_{1} }}} \right)\left( {\frac{{\partial^{2} \psi }}{{\partial y^{2} }}} \right)^{2} }}_{{N_{F} }} + \underbrace {{A_{3} \frac{{BrM^{2} }}{{\Omega }}\left( {1 + \frac{\partial \psi }{{\partial y}}} \right)^{2} }}_{{N_{J} }} + \underbrace {{\left( {\frac{{\Pi }}{{\Omega }}\frac{{\partial {\Theta }}}{\partial y}} \right)^{2} + \frac{{\Pi }}{{{\Omega }Le}}\left( {\frac{\partial \theta }{{\partial y}}\frac{{\partial {\Theta }}}{\partial y}} \right)}}_{{N_{M} }},$$

where ${N}_{T}$ is the thermal irreversibility, ${N}_{V}$ is the viscous irreversibility, ${N}_{F}$ the fluid friction irreversibility, ${N}_{J}$ is the Joule heating irreversibility, and ${N}_{M}$ is the mass transfer irreversibility. The ratio between irreversibility caused by heat transfer and total irreversibility caused is given by Bejan number, Be. It is defined in mathematical form:

30 $$Be=\frac{{A}_{5}{\left(\frac{\partial \theta }{\partial y}\right)}^{2}}{{A}_{5}{\left(\frac{\partial \theta }{\partial y}\right)}^{2}+\frac{Br}{\Omega }{\left(\frac{{\partial }^{2}\psi }{\partial {y}^{2}}\right)}^{2}+{A}_{2}\frac{Br}{\Omega }\left(\frac{1}{1+{\lambda }_{1}}\right){\left(\frac{{\partial }^{2}\psi }{\partial {y}^{2}}\right)}^{2}+{A}_{3}\frac{Br{M}^{2}}{\Omega }{\left(1+\frac{\partial \psi }{\partial y}\right)}^{2}+{\left(\frac{\Pi }{\Omega }\frac{\partial\Theta }{\partial y}\right)}^{2}+\frac{\Pi }{\Omega Le}\left(\frac{\partial \theta }{\partial y}\frac{\partial\Theta }{\partial y}\right)}.$$

## Numerical solution

The model’s governing Eqs. (19, 20, 21, 22, 23) are a set of coupled, highly nonlinear ordinary differential equations. The gunshot method is used to resolve this system of differential equations. By turning a boundary value problem into an initial value problem of a first order differential equation, the shooting method can solve a BVP. It entails searching for IVP solutions that also satisfy the boundary conditions of the BVP for a variety of beginning conditions. A practical shooting approach is used to solve the governing differential Eqs. (19, 20, 21, 22, 23) and the accompanying boundary conditions (24). Here are the substitutions that are used:

31 $$\begin{aligned} & {Y}_{1}=\psi , {Y}_{2}=\frac{\partial \psi }{\partial y}, {Y}_{3}=\frac{{\partial }^{2}\psi }{\partial {y}^{2}}, {Y}_{4}=\frac{{\partial }^{3}\psi }{\partial {y}^{3}}, {Y}_{5}= \theta , {Y}_{6}=\frac{\partial \theta }{\partial y}, {Y}_{7}=\Theta , {Y}_{8}=\frac{\partial\Theta }{\partial y},\\ & {Y}_{9}=\frac{\partial \mathrm{\varphi }}{\partial y}, {Y}_{10}=\frac{{\partial }^{2}\varphi }{\partial {y}^{2}}, {Y}_{6}{^\prime}={\xi }_{1}, {Y}_{8}{^\prime}={\xi }_{2}, {Y}_{10}{^\prime}={\xi }_{3}.\end{aligned}$$

These substitutions result in an initial value problem consisting of nine first-order differential equations:

32 $$\left.\begin{array}{c}{Y}_{1}{^\prime}={Y}_{2},\\ {Y}_{2}{^\prime}={Y}_{3},\\ {Y}_{3}{^\prime}={Y}_{4},\\ {Y}_{4}{^\prime}=\frac{\left[{A}_{3}{M}^{2}+\frac{{A}_{2}}{Da}+2ReFr\left(1+{Y}_{2}\right)\right]{Y}_{3}-{A}_{1}Gr{\mathrm{Y}}_{6}\mathrm{cos}\eta +Gc{\mathrm{Y}}_{8}\mathrm{cos}\eta -{M}_{f}\left({Y}_{10}^{2}+{Y}_{9}{\xi }_{3}\right)}{\left({A}_{2}+\frac{1}{1+{\lambda }_{1}}\right)},\\ {Y}_{5}{^\prime}={Y}_{6},\\ {Y}_{6}{^\prime}=\frac{-Br\left[{A}_{2}{\left({Y}_{3}\right)}^{2}\right]-PrNb{Y}_{6}{Y}_{8}-PrNt{\left({Y}_{6}\right)}^{2}-Q{Y}_{5}{e}^{-y}-PrK{r}_{1}K{r}_{2}{Y}_{7}{\left(1+\Omega {Y}_{5}\right)}^{n}{e}^{-\left(\frac{{E}^{*}}{1+\Omega {Y}_{5}}\right)}}{{A}_{5}},\\ {Y}_{7}{^\prime}={Y}_{8},\\ {Y}_{8}{\prime}=-\frac{Nt}{Nb}{\xi }_{2}+K{r}_{1}Sc{Y}_{7}{\left(1+\Omega {Y}_{5}\right)}^{n}{e}^{-\left(\frac{{E}^{*}}{1+\Omega {Y}_{5}}\right)},\\ {Y}_{9}{^\prime}={Y}_{10},\\ {Y}_{10}{^\prime}=-P{r}_{m}Re{Y}_{9}{Y}_{3}\end{array}\right\}.$$

With the following boundary conditions:

33 $${Y}_{1}\left(-h\right)=\frac{F}{2};{Y}_{2}\left(-h\right)=-1;{Y}_{5}\left(-h\right)=0;{Y}_{7}\left(-h\right)=0;{Y}_{9}\left(-h\right)=0;{Y}_{1}\left(+h\right)=-\frac{F}{2};{Y}_{2}\left(+h\right)=-1;{Y}_{5}\left(+h\right)=1;{Y}_{7}\left(+h\right)=1;{Y}_{10}\left(+h\right)=0.$$

## Results validation

**Figure 2:**
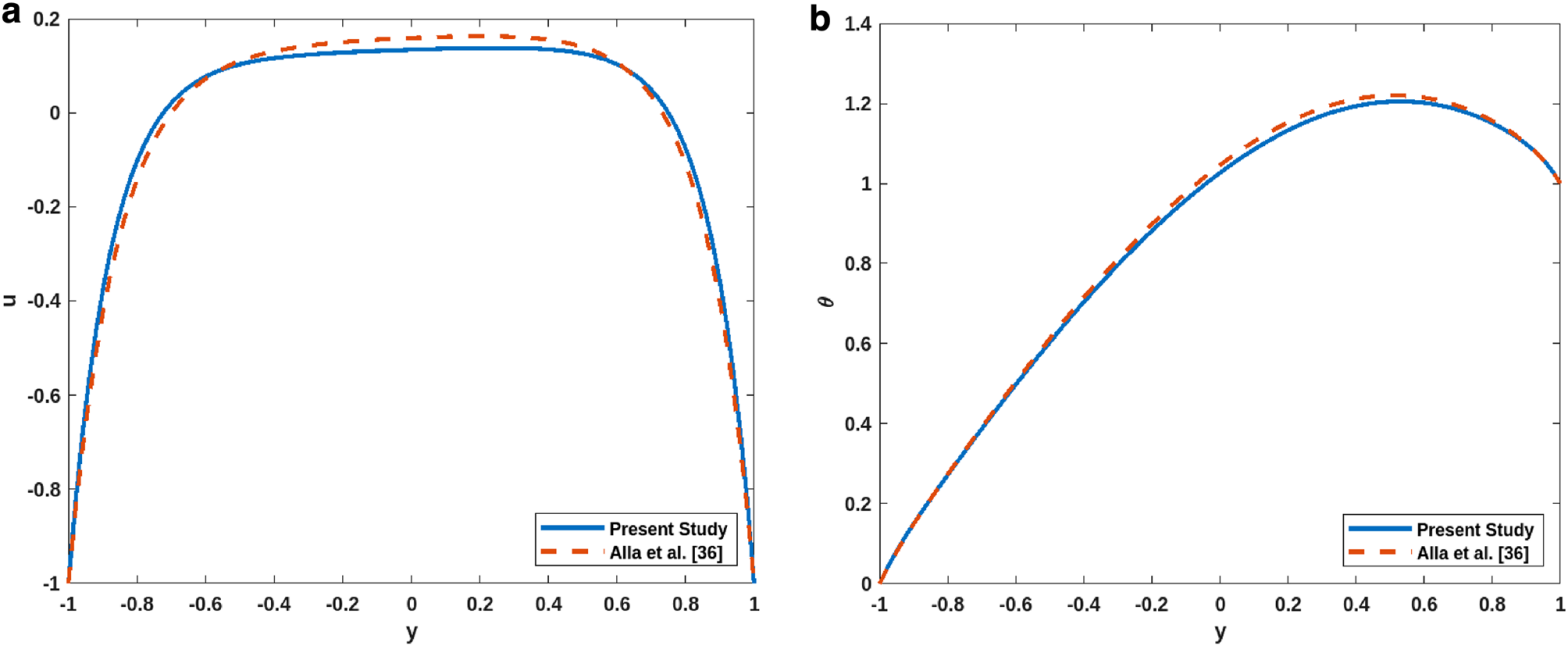
(**a**) Comparative analysis of $\mathrm{u}$. (**b**) Comparative analysis of $\uptheta$.

Figure 2a,b show a comparative study of velocity $u$ and temperature $\theta$. The present model is reduced to an already published work of Alla et al.^36^. Figure 2a,b illustrate the velocity and temperature profiles for a combination of dimensionless parameters, respectively. It can be concluded from both figures that the present study is in accordance with the earlier published work. Table 2 shows the range of the values of parameter and corresponding references of parameters.

**Table 2:** Ranges of the values of flow parameters.

Parameter	$Gr$	$Gc$	$Re$	$M$	$Da$	$P{r}_{m}$	$Br$	$Q$	$F$	$\phi$	$Fr$
Range	[0–8]	[0–8]	[0–8]	[0–10]	[0–1]	[0–20]	[0–5]	[− 10,10]	[0–1]	[0–1]	[0–20]
Default	0.5	0.5	0.5	2	0.03	0.5	0.5	0.5	0.05	0.1	0.5
References	^55^	^55^	^55^	^50^	^56^	^57^	^52^	^51^	^58^	^54^	^56^

Parameter	${\lambda }_{1}$	$n$	${M}_{f}$	$\Omega$	$Sc$	$Nb$	$Nt$	$K{r}_{1}$	$K{r}_{2}$	$E$	
Range	[0–5]	[0–1]	[0–20]	[0–1]	[0–10]	[0–5]	[0–5]	[0–15]	[− 10,10]	[− 10,10]	
Default	1	1	2	1	0.3	0.5	0.5	0.5	0.5	1	
References	^36^	^40^	^57^	^53^	^53^	^53^	^53^	^40^	^40^	^40^	

The numerical results for Eq. (32) with the boundary conditions (33), are obtained using MATLAB’s BVP5C programme.

## Results and discussions

A combined effect of external and induced magnetic field, viscous dissipation, highly porous medium, thermophoresis, Brownian motion, exponential heat source/sink, activation energy, and endothermic-exothermic chemical reactions are scrutinized in this section. For a given set of flow parameters, various graphs of velocity, temperature, concentration, and induced magnetism profiles are drawn.

### Velocity profiles

**Figure 3:**
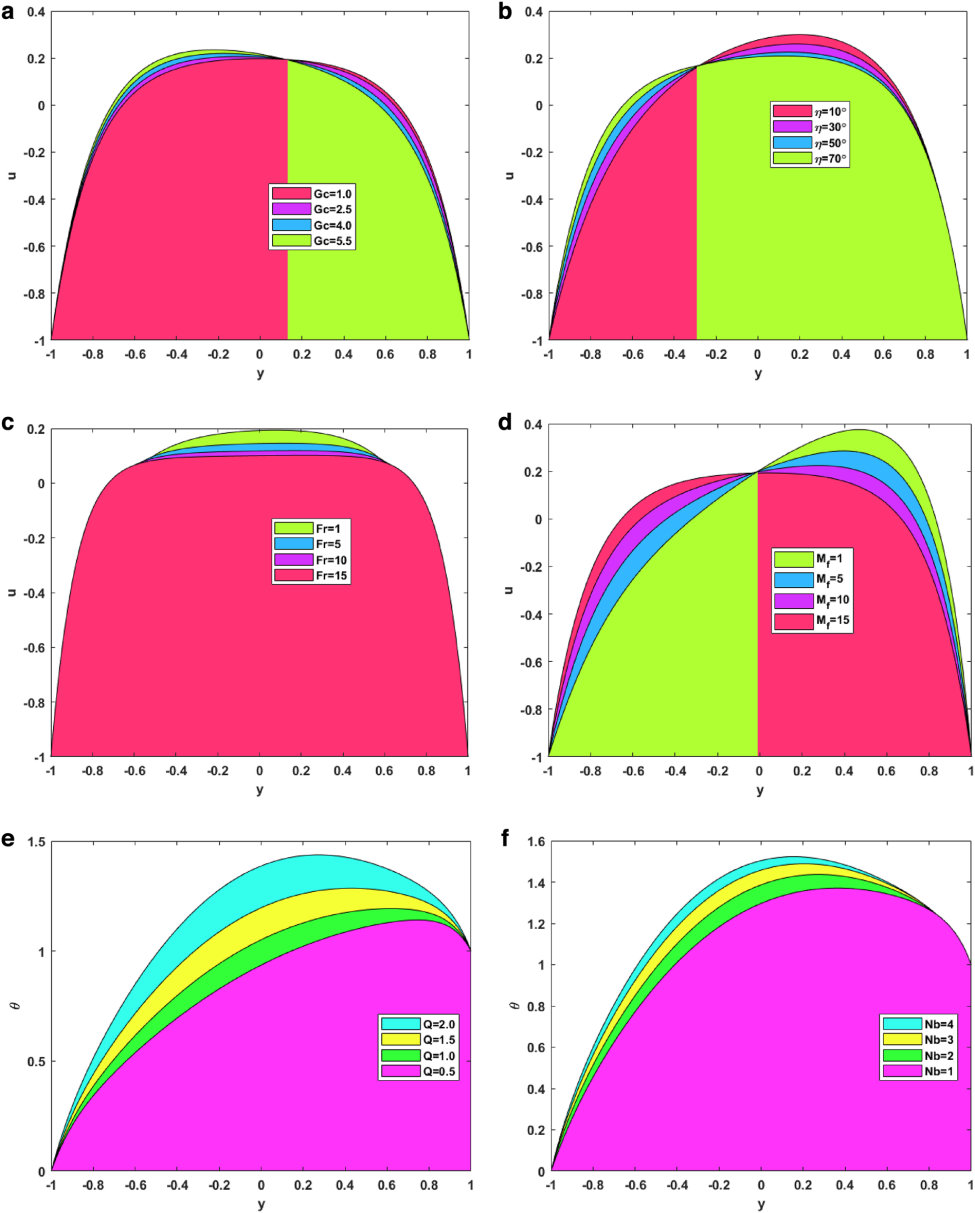

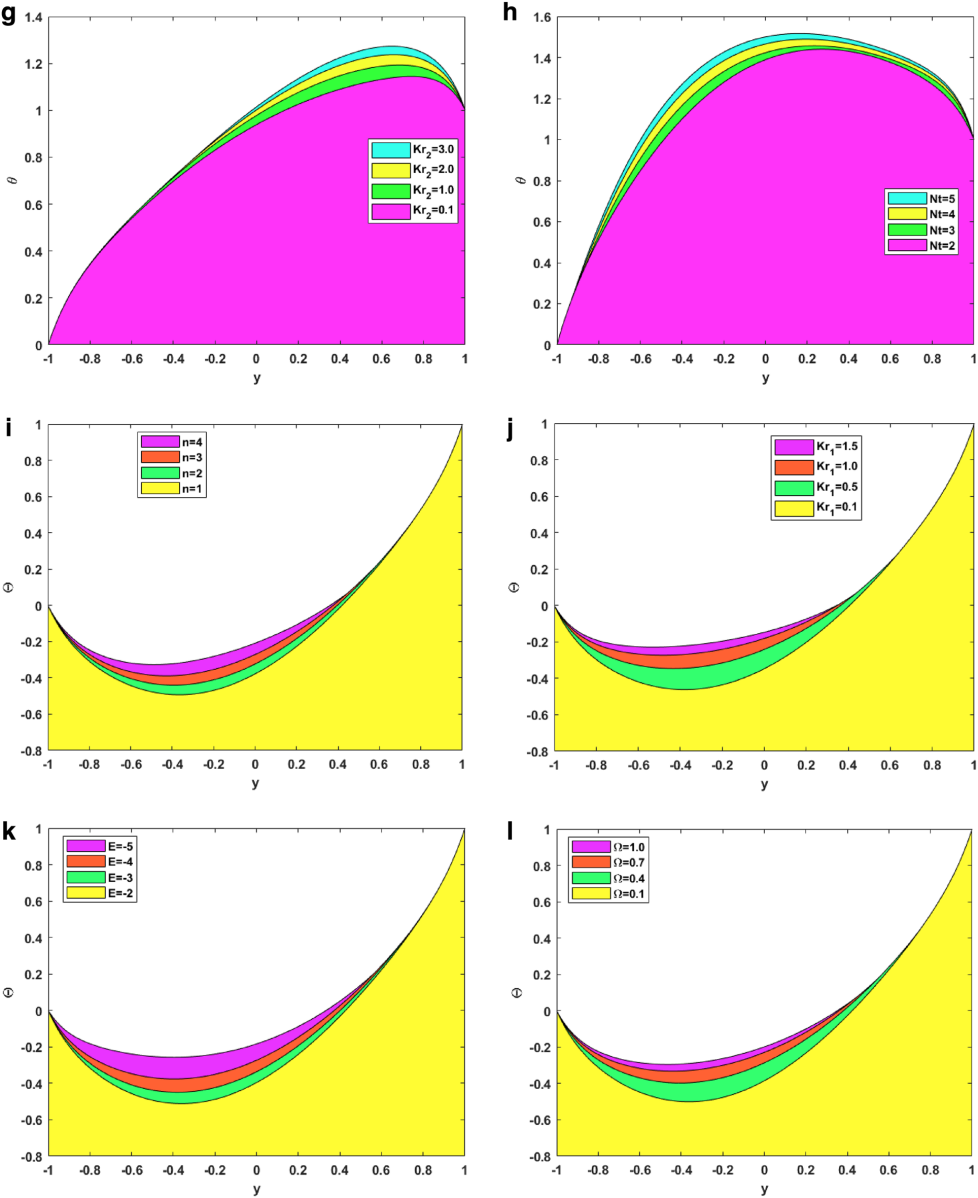

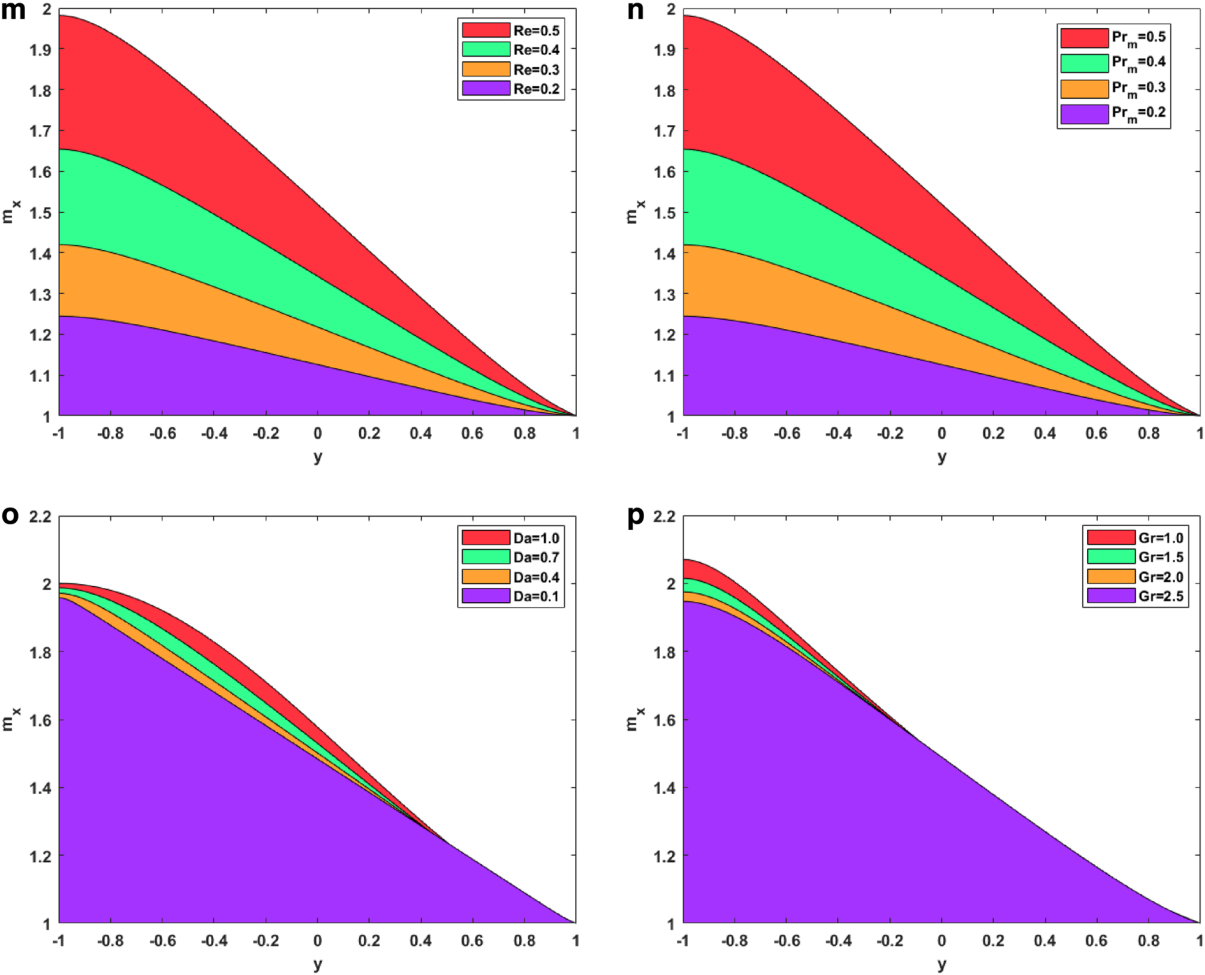
(**a**) Velocity VS Thermal Grashof number. (**b**) Velocity VS channel inclination. (**c**) Velocity VS Forchheimer number. (**d**) Velocity VS induced magnetic field number. (**e**) Temperature VS Heat source number. (**f**) Temperature VS Brownian motion number. (**g**) Temperature VS endo-exo reaction number. (**h**) Temperature VS thermophoresis number. (**i**) Concentration VS fitting constant. (**j**) Concentration VS chemical reaction number. (**k**) Concentration VS activation energy number. (**l**) Concentration VS temperature ratio. (**m**) Induced magnetism VS Reynolds number. (**n**) Induced magnetism VS magnetic Prandtl number. (**o**) Induced magnetism VS Darcy number. (**p**) Induced magnetism VS thermal Grashof number.

Figure 3a–d illustrate the impact of the solutal Grashof number, channel inclination, Forchheimer parameter, and induced magnetic field number on the axial velocity of the fluid. The link between the solutal Grashof number and the fluid’s velocity is depicted in Fig. 3a. It is noted that, for enhanced values of solutal Grashof number, the axial velocity first increase and then decrease. The change happens at almost y = 0.1. The ratio of a species' buoyancy to viscous force is physically represented by the solutal Grashof number. The values in the figure are all greater than 1, meaning that the buoyancy force’s effect is larger than the viscous force. Figure 3b shows how increasing the channel inclination affects the fluid velocity. The figure depicts that for raised values of inclination, the axial velocity increases for the first half and then decreases for the second half. When the inclination increases, the magnetic field’s effect dominates that of gravity. As the magnetic field acts perpendicular to the channel, the velocity increases for the fallen magnetic strength. For the second half, gravitational acceleration dominated the magnetic field, and on increasing the inclination, the gravitational acceleration dropped the speed. Figure 3c depicts the effect of the Forchheimer number on the axial velocity. It can be seen in the figure that for raised values of the Forchheimer number, the velocity decreases. Increasing the Forchheimer number decreases fluid velocity since the inertia factor is directly correlated with the medium’s porosity and drag coefficient. As a result, as the Forchheimer number rises, so does the drag coefficient and the porosity of the medium. A greater Forchheimer number is associated with lower velocities since the liquid’s resistive force increases with decreasing speed. Figure 3d explains the importance of the induced magnetic field on fluid velocity. The figure signifies that for enhanced values of induced magnetic field number, the velocity first increases and then decreases. This change happens at y = 0. Induced magnetism happens in nanofluids when an external magnetic field causes nanoparticles suspended in a base fluid to take on magnetic characteristics. The nanoparticles’ magnetic moments can align with the magnetic field, resulting in a net magnetic moment in the fluid, which causes this effect.

### Temperature profiles

Figure 3e–h signifies the effect of the heat source number, Brownian motion parameter, endothermic–exothermic chemical reaction number, and the thermophoresis number on the fluid’s temperature profiles. The effect of the heat source parameter on the temperature profile is seen in Fig. 3e. The figure shows that the temperature increases when the heat source strength increases. From the physical point of view, these results make sense, as enhanced heat source strength implies more heat generation from the region’s surface. Figure 3f illustrates how the Brownian motion parameter affects temperature. The figure demonstrates that the temperature profiles also increase for increased values of Brownian motion parameters. The fluid’s swiftly moving atoms and molecules, have a greater influence on the Brownian motion of the suspended particles in the base fluid. The relationship between Brownian motion and particle size and the fact that these particles commonly assume the form of aggregates or agglomerates should be noted. For large particles, the strength of Brownian motion is minimal, leading to low values for the parameter. As the values of the Brownian motion parameter increase, so do the temperature profiles in the boundary layer region. The purpose of Fig. 3g is to depict the effect of enhanced endothermic-exothermic chemical reaction parameters on the temperature profiles. The figure shows that the temperature profiles improve for enhanced parameter values. This parameter takes negative values for an exothermic reaction, and for an endothermic reaction, it is positive. The figure shows the positive values or the endothermic nature of the reaction. During an endothermic reaction, the nanoparticles absorb energy from the surroundings to overcome the activation energy barrier. The surrounding temperature decreases, and the fluid temperature increases. The importance of thermophoresis numbers on the temperature profiles is shown in Fig. 3h. The figure signifies that the temperature profiles increase by elevating the thermophoretic effect. It has been demonstrated that temperature profiles are enhanced when the thermophoretic parameter has higher values. This occurs due to the thermophoretic force that particle adjacent to a hot surface produce. By increasing the thickness of the temperature boundary layer, this force encourages particle disintegration outside of the fluid regime.

### Concentration profiles

Figure 3i–l represents the impact on the fluid concentration for fitting constant, chemical reaction number, activation energy, and temperature ratio. Figure 3i depicts the consequence of smaller and larger fitting constants on the temperature. For larger fitting constant, the concentration profiles improve in the positive direction. The temperature fitting constant is used to adjust the thermal conductivity and viscosity of the nanofluid to account for these differences. Figure 3j shows the aftermath of increased chemical reaction parameters on the concentration profiles. The figure shows that for enhanced values of chemical reaction parameters, the concentration profiles of fluid move in the upper direction. Increases in the concentration of the ternary hybrid nanofluid and the thickness of the boundary layer result from an increase in the chemical reaction parameter close to the surface. It is thought to increase the likelihood of collisions between fluid particle atoms. Figure 3k indicates the concentration profile behavioral change for the activation energy parameter. An increased behavior is seen in the concentration profiles for escalated activation energy because the activation energy is regarded as a strong potential barrier separating the potential energy minima. Because activation energy supplies the energy to initiate the chemical reaction, increasing concentration. Because of amplification in activation energy, which supplies significant energy to process the reaction and increase the concentration field, the concentration profile grows. Figure 3l is used to demonstrate the temperature ratio changes in the concentration. The concentration parameter for the expanding temperature ratio exhibits an incline behavior. This phenomenon occurs because the parameter describing the thermal state of a fluid, the temperature ratio, increases as the temperature rises. It describes the fluid’s thermal condition; as this parameter rises, so does the temperature.

### Induced magnetism profiles

Figure 3m–p represents the changes in induced magnetic field behavior for the Reynolds number, magnetic Prandtl number, Darcy number, and thermal Grashof number. Figure 3m signifies the changes in the Reynolds number on the induced magnetism. The figure symbolizes that the induced magnetism profiles increase by increasing the Reynolds number. This is because viscosity and the Reynolds number are inversely correlated. As the Reynolds number rises, viscosity falls, making the fluid less thick and increasing velocity. Conversely, viscosity rises as the Reynolds number falls, making the fluid thicker and decreasing velocity. For enhanced velocity, the fast-moving nanoparticles induce much greater magnetism. Figure 3n signifies the changes in magnetic Prandtl number on the induced magnetic field. The figure shows how the induced magnetism increases on increasing the magnetic Prandtl number. Physically, the magnetic Prandtl number is proportional to the heat transfer rate. For larger values of magnetic Prandtl number, the temperature and Brownian motion of nanoparticles increase, which eventually increases the velocity. Again, for more velocity, more magnetism is induced in the fluid. Figure 3o depicts the effect of enhanced Darcy number. It shows that the induced magnetic field rises for a larger Darcy number. This is due to the fact that the medium becomes more permeable at higher Darcy values, which in turn creates more room for nanofluid recirculation and a greater velocity. Once more, greater velocity creates more magnetism. Figure 3p shows the significance of the larger thermal Grahsof number on the induced magnetism profile of the fluid. On enhancing the thermal Grashof number, the fluid developed more induced magnetism. Physically, the thermal Grashof number indicates the ratio of buoyancy force to the viscous force. The viscous forces become lower for incremented values of the thermal Grashof number. These lower viscous forces increase the fluid velocity, eventually increasing the fluid’s induced magnetism.

### Velocity contour plots

**Figure 4:**
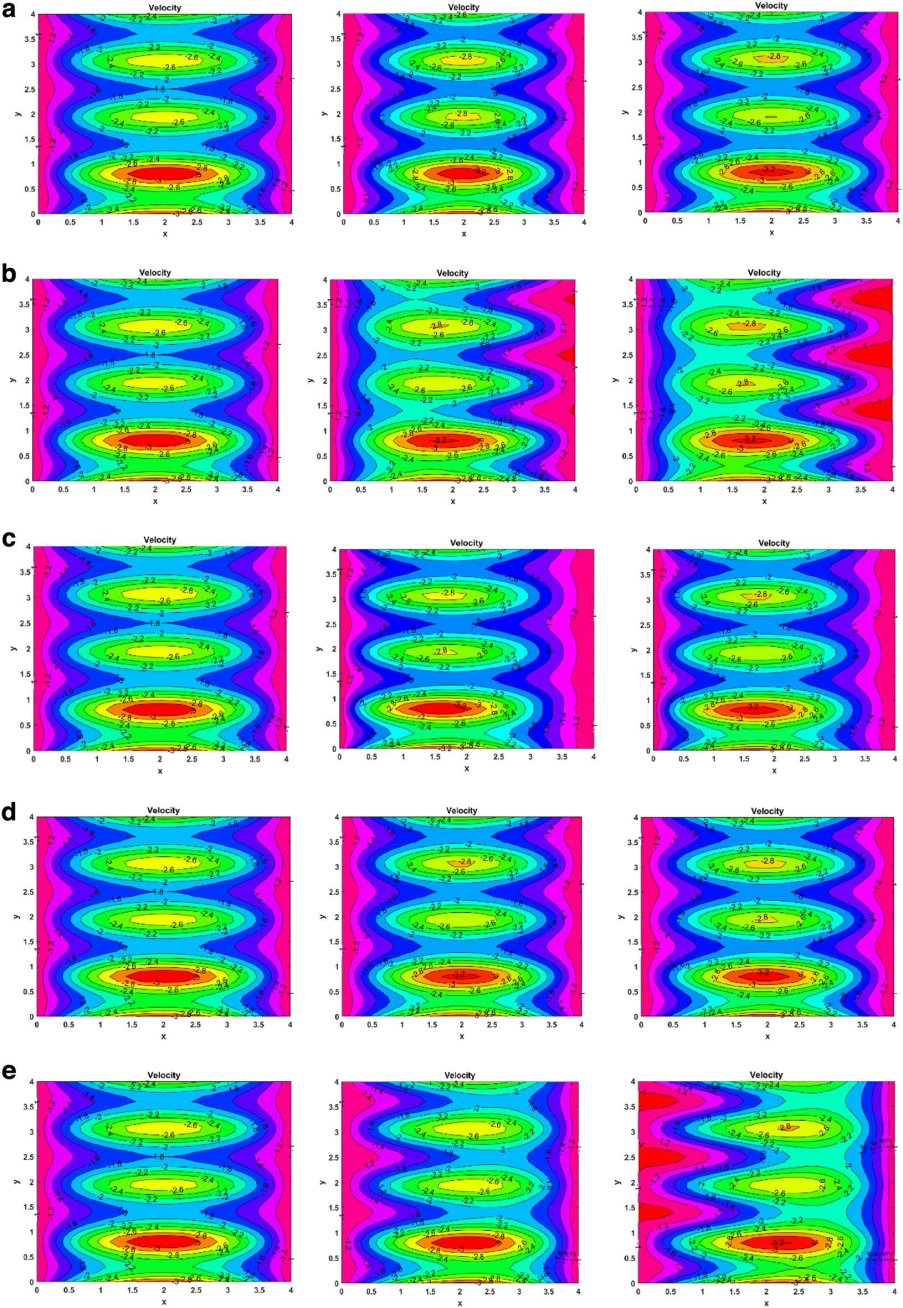
(**a**) Velocity contours for Forchheimer number (Fr = 1, 5, 10). (**b**) Velocity contours for Solutal Grashof number (Gc = 1, 4, 8). (**c**) Velocity contours for Induced magnetic field number (M_f_ = 2, 8, 14). (**d**) Velocity contours for Reynolds number (Re = 1, 2.5, 5). (**e**) Velocity contours for Thermal Grashof number (Gr = 1, 4, 8).

Figure 4 shows the contour plots for fluid’s velocity for solutal and thermal Grashof number, induced magnetic field number, Forchheimer number, and Reynolds number. Figure 4a illustrates the effect of the Forchheimer number on the velocity. By increasing Fr from 1 to 5, the size of the innermost yellow bubble decreases. The overall size of all the bubbles decreases for the flow. An annulus is also formed at the upper part of the channel. On making Fr 10, a smaller orange-coloured bolus is formed at the top. The annular region of the lower part also decreased. Small bubbles can be seen in the middle portion of the flow. Figure 4b shows the velocity contour behaviour for increasing the solutal Grashof number. By increasing Gc from 1 to 4, the rightward waves become sharper. The bubbles become narrower from the right side. There is also a formation of orange bolus in the topmost annulus. For the Gc value of 8, the waves become more sharer at the right side. The formation of orange bolus in the upper two annuli enhanced. The next Fig. 4c, demonstrates how the changing induced magnetic field impacts the velocity. The figure shows that the innermost yellow bubbles become smaller when the induced magnetism parameter increases from 2 to 8. In the middle annulus, it got disappeared. For the Mf value of 14, the inner bolus again starts emerging. One can see an orange-coloured bubble in the middle of the upper bolus. The purpose of Fig. 4d is to show how increasing the Reynolds number changes the flow velocity. On making Re as 2.5, it can be seen that the size of the inner bolus becomes smaller. There is also a formation of 2.8 valued bolus in the uppermost annulus. For the Re value of 5, the formation of boluses again starts, but now of higher value (in the negative direction). Figure 4e represents the thermal Grashof number vs the velocity contour. It shows that when Gr is changed to 4 from 1, waves are leftward squeezing. The wavelength decreases, and the amplitude increases drastically. For the Gr value of 8, there is again a significant jump in amplitude is seen. There is also a formation of 2.8 valued bolus in the uppermost annulus.

### Velocity surface plots

**Figure 5:**
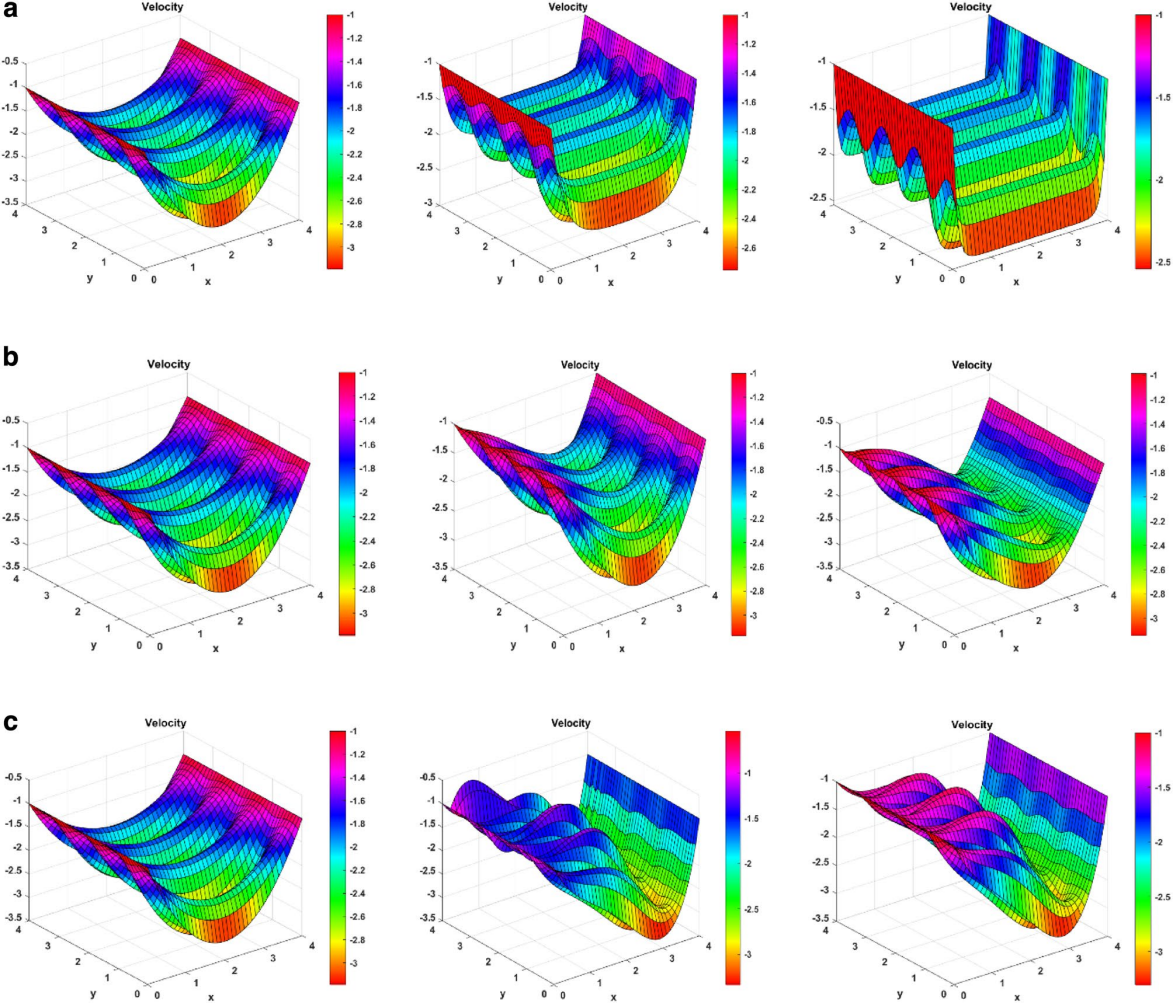
(**a**) Velocity surface plots for Darcy number (Da = 0.1, 0.5, 1). (**b**) Velocity surface plots for Solutal Grashof number (Gc = 1, 4, 8). (**c**) Velocity surface plots for Magnetic number (M = 1, 5, 10).

Figure 5 shows the velocity surface plots for Darcy, solutal Grashof, and magnetic field number. Figure 5a shows how changing Darcy number affects the fluid velocity. On raising the Darcy number, the velocity decreases. Physically, as the medium porosity increases, the fluid experiences more resistance for its motion. For the increased value of Darcy number, there is an upward shift of the plots, meaning that the velocity increased. An increasing effect is seen on the left and right sides of the flow, meaning the boundary nanoparticles also boost their velocity. Figure 5b shows the effect of the solutal Grashof number. It says that on raising the values, the velocity increases. Physically, the Grashof number is inversely proportional to the viscous forces. When these forces decrease, the velocity increases. The plot shows a better velocity increase for the leftward nanoparticles. There is more elevation in the leftward waves, and the rightward waves also suffer a lower shift. The next Fig. 5c, shows the effect of magnetic field number. When the magnetic field number is increased, the Lorentz force is developed. This force decreases the fluid velocity. The figure shows that for increased M values, the plots shift downward, meaning a decrease in the velocity. The right side sees a good velocity drop. However, the left wave experiences a lower drop than the other side.

### Temperature surface plots

**Figure 6:**
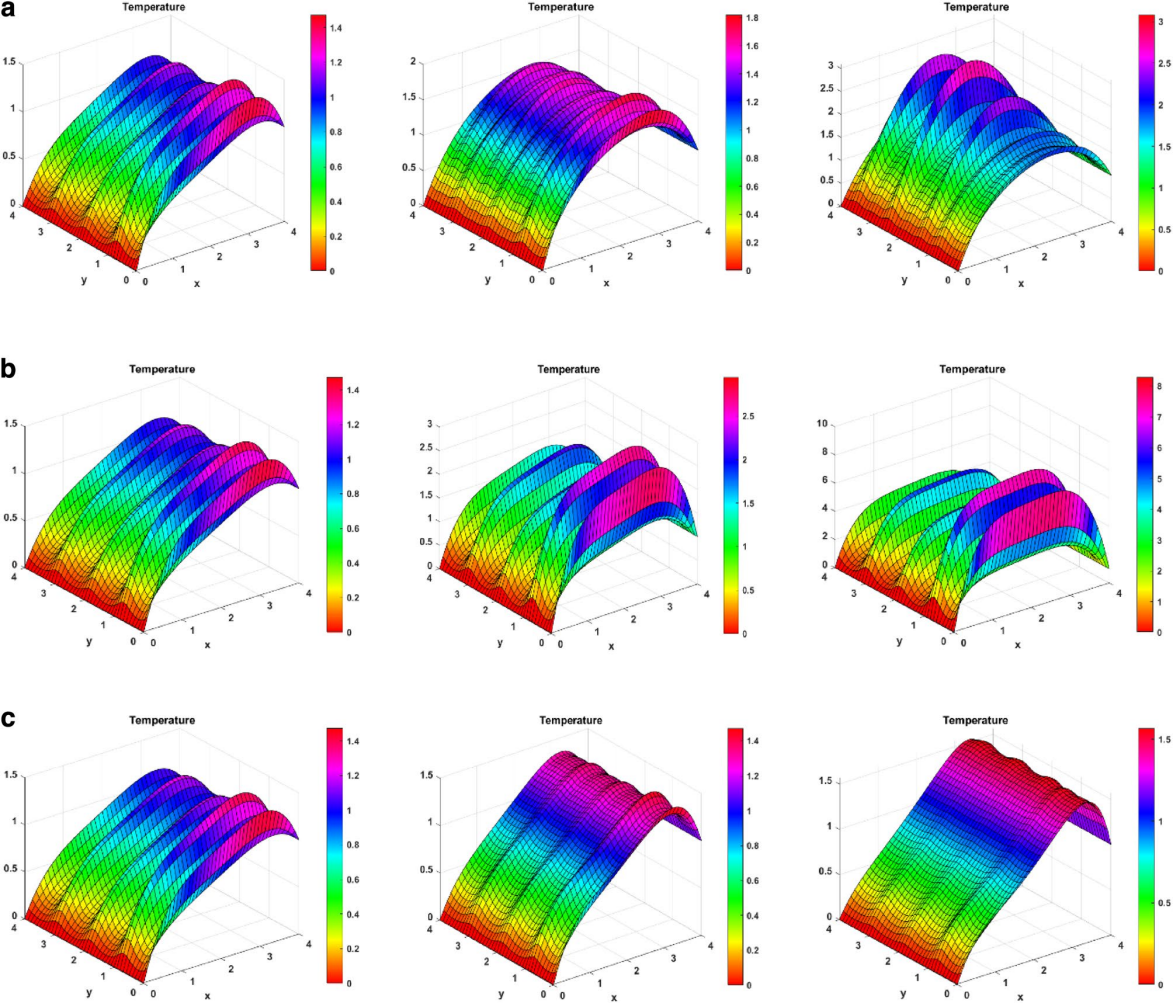
(**a**) Temperature surface plots for Heat source number (Q = 1, 2.5, 5). (**b**) Temperature surface plots for Brinkman number (Br = 1, 2.5, 5). (**c**) Temperature surface plots for endothermic–exothermic reaction parameter (Kr_2_ = 1, 5, 10).

Figure 6 shows the effect on temperature behaviour for the heat source, Brinkman, and endothermic–exothermic chemical reaction parameters. Figure 6a shows the increasing change of heat source number on temperature. As the heat source number increases, more heat energy is released to the fluid and the fluid’s temperature increases. For enhanced values of heat source, the temperature plots experience an upward shift, representing an increase in temperature. For a Q value of 5, there is a good jump in temperature at later y-values. Figure 6b expresses how the increasing Brinkman number affects the temperature. For elevated Brinkman number, the kinematic collisions rise, and the entropy rises. This result can be seen for lower y-values with rising temperature curves. However, for higher y-values, the effect does not dominate in the presence of other factors, and a temperature decrease is seen. Figure 6c shows the change of endothermic–exothermic reaction parameter on temperature. As the values for this parameter increase, the temperature also increases. The plots show an upward shift of curves for enhanced Kr2.

### Concentration surface plots

**Figure 7:**
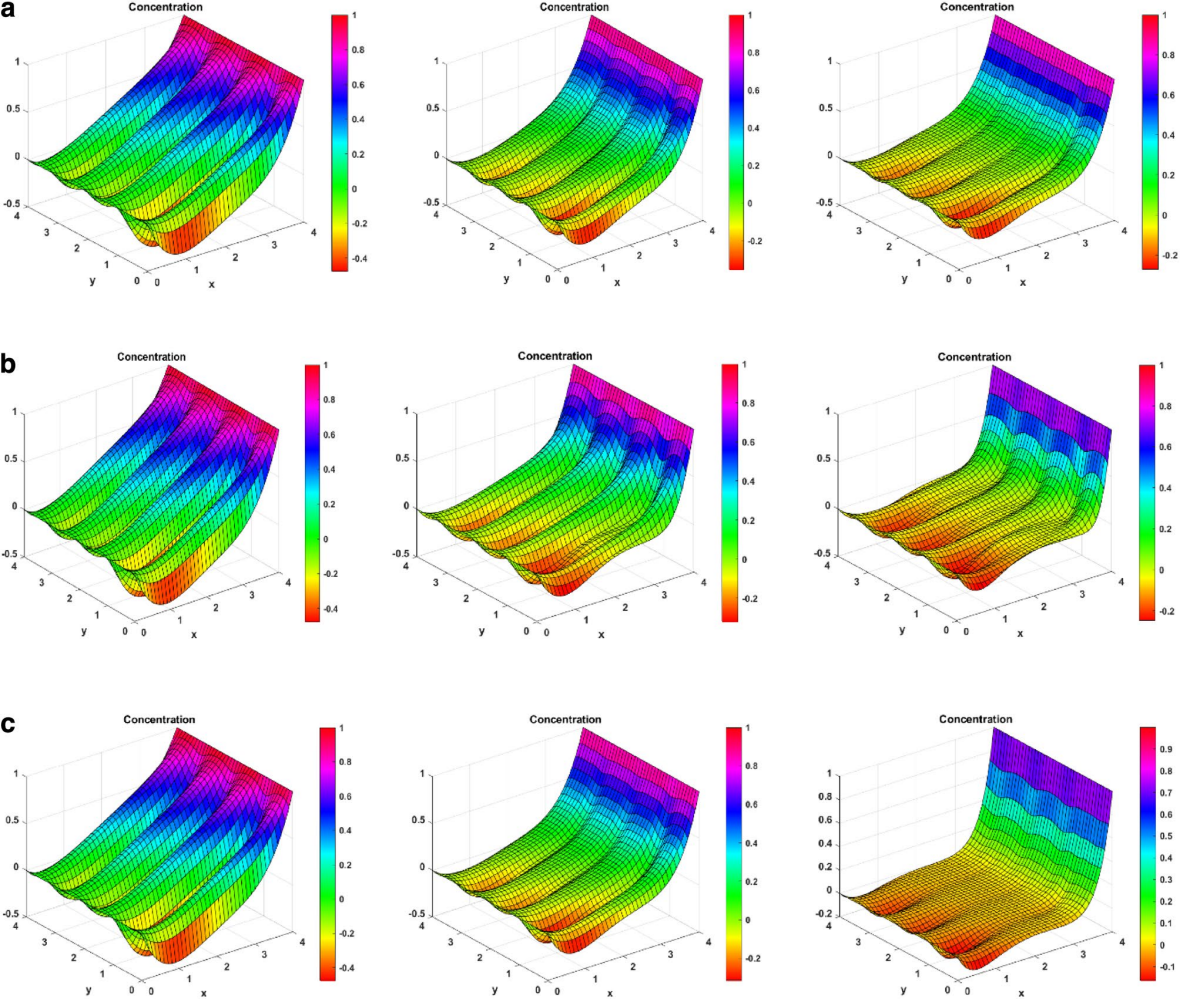
(**a**) Concentration surface plots for Chemical reaction parameter (Kr_1_ = 1, 3, 5). (**b**) Concentration surface plots for Fitting constant (n = 0.1, 0.5, 1). (**c**) Concentration surface plots for Temperature ratio ($\Omega$ = 0.1, 0.5, 1).

Figure 7 represents the 3D surface plots for concentration for fitting constant, chemical reaction parameter, and temperature ratio. Figure 7a first shows the changes in chemical reaction numbers on the concentration curves. The figure depicts that the nanoparticles’ collisions rise for enhanced chemical reaction parameters, decreasing their concentration. As the chemical reaction parameter grows, the concentration plots move downward. For higher x-values, there is a significant difference seen for concentration differences. Now, Fig. 7b reveals the impact of fitting constant on the concentration curves. For these fitting constant values, a concentration drop is seen for higher x-values. But for lower x-values, the reverse effect is seen. In the middle of the channel, an increase in concentration curves is seen again. Figure 7c shows the temperature ratio change on the concentration curves. The figure demonstrated that for increased temperature ratio values, the concentration drops for higher x-values. For lower x-values and in the middle of the channel, concentration increases.

### Induced magnetism surface plots

**Figure 8:**
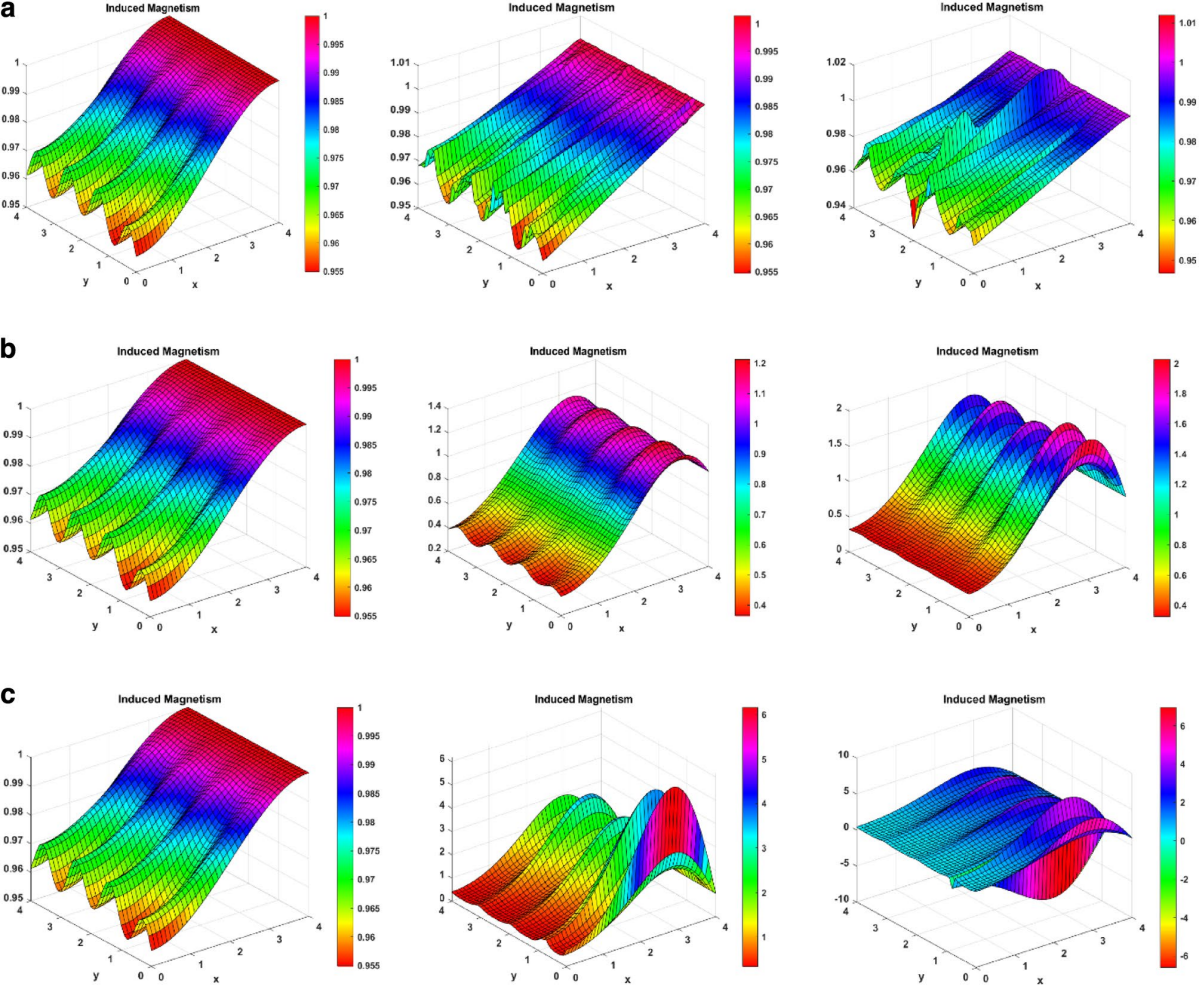
(**a**) Induced magnetism surface plots for magnetic number (M = 1, 5, 10). (**b**) Induced magnetism surface plots for magnetic Prandtl number (Pr_m_ = 1, 2.5, 5). (**c**) Induced magnetism surface plots for Reynolds number (Re = 0.2, 0.4, 0.6).

Figure 8 reveals the impact on induced magnetism for increasing magnetic, magnetic Prandtl, and Reynolds numbers. Figure 8a reveals that for enhanced values of the magnetic number, the Lorentz forces start developing on the fluid. In response to these forces, lesser magnetism is induced for higher y-values. However, for lesser y-values, there is a good magnetism development in the fluid. The middle portion of the channel also sees a jump at the peaks of the curves. Figure 8b represents the enhanced behavior for magnetic Prandtl number and induced magnetism in the fluid. The magnetic Prandtl number is inversely proportional to the velocity of the fluid. For larger Pr_m_, the induced magnetism decreases for the right side of the channel. For the left waves of the channel, the induced magnetic field almost remains the same, or a little increment is seen. Furthermore, Fig. 8c is made for the Reynolds number and induced magnetism. The figure depicts that when the Reynolds number is increased, there is a higher shift of magnetism for the middle part of the channel, as the Reynolds number is inversely proportional to the viscous forces. As these forces decrease, the velocity increases, increasing the inducing effect. The Y-axis’s higher part shows a slight decrease in the induced magnetism effect.

### Correlation matrices

The correlation coefficient expresses the degree of association between two or more variables. It indicates the strength of the relationship between two variables, as well as the direction of the relationship (positive or negative). Typically, correlation is depicted by a coefficient ranging from − 1 to 1. A perfect positive correlation is represented by a correlation coefficient of 1, whereas a perfect negative correlation is represented by a correlation coefficient of 1. If the coefficient is 0, there is no association between the two variables. The formula for the correlation coefficient (Pearson’s $r$) between two variables p and q can be written as:

$$r=\frac{{\sum }_{i=1}^{n}\left({p}_{i}-\overline{p }\right)\left({q}_{i}-\overline{q }\right)}{\sqrt{{\sum }_{i=1}^{n}{\left({p}_{i}-\overline{p }\right)}^{2}}\sqrt{{\sum }_{i=1}^{n}{\left({q}_{i}-\overline{q }\right)}^{2}}},$$

where $n$ is the size of sample used in the study, ${p}_{i}$ and ${q}_{i}$ are the values of p and q for the ith observation, $\overline{p }$ and $\overline{q }$ are the sample means of p and q, respectively.

**Figure 9:**
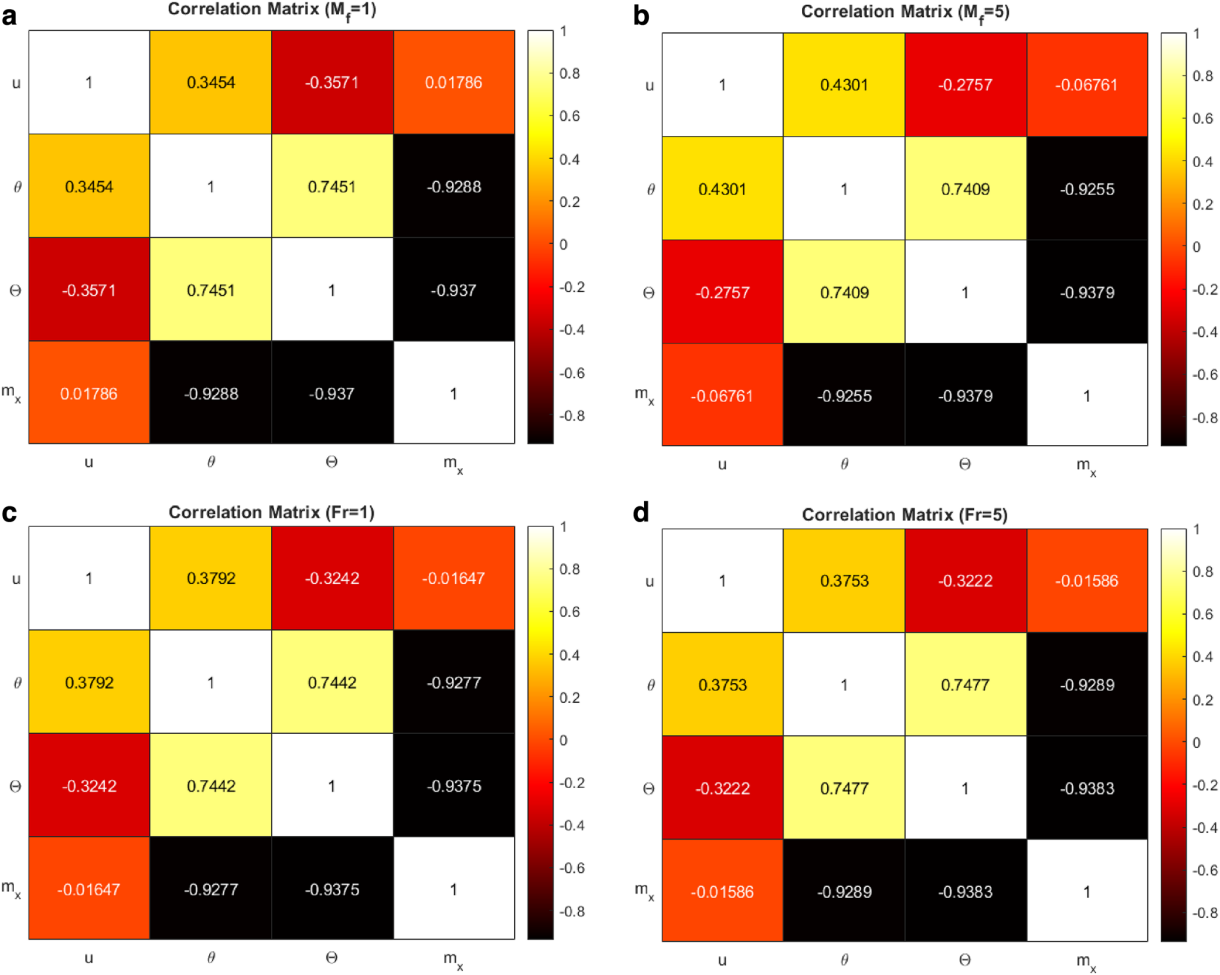
(**a**) Correlation matrix for M_f_ = 1. (**b**) Correlation matrix for M_f_ = 5. (**c**) Correlation matrix for Fr = 1. (**d**) Correlation matrix for Fr = 5.

Figure 9a,b show the correlation matrix for M_f_ = 1 and M_f_ = 5. When the induced magnetic field number rises, the positive correlation between the velocity and the temperature increases. The negative correlation between the velocity and concentration decreases as M_f_ increase. The velocity and induced magnetism correlation become negative from positive. The temperature and concentration positive correlation decreases. The negative correlation between temperature and induced magnetism decreases. The concentration and induced magnetic field increase in the positive direction for the third decimal place. Figure 9c,d represent the correlation matrix for Fr = 1 and Fr = 5. On increasing the Forchheimer number, the positive correlation between velocity and temperature decreases. The negative correlation of velocity and concentration decreases in the negative direction. The negative correlation of velocity and induced magnetic field drops. The positive correlation between temperature and concentration enhances. The negative correlation between temperature and induced magnetism rises. Concentration and induced magnetism correlation increases in the positive direction.

### Entropy generation and Bejan number

**Figure 10:**
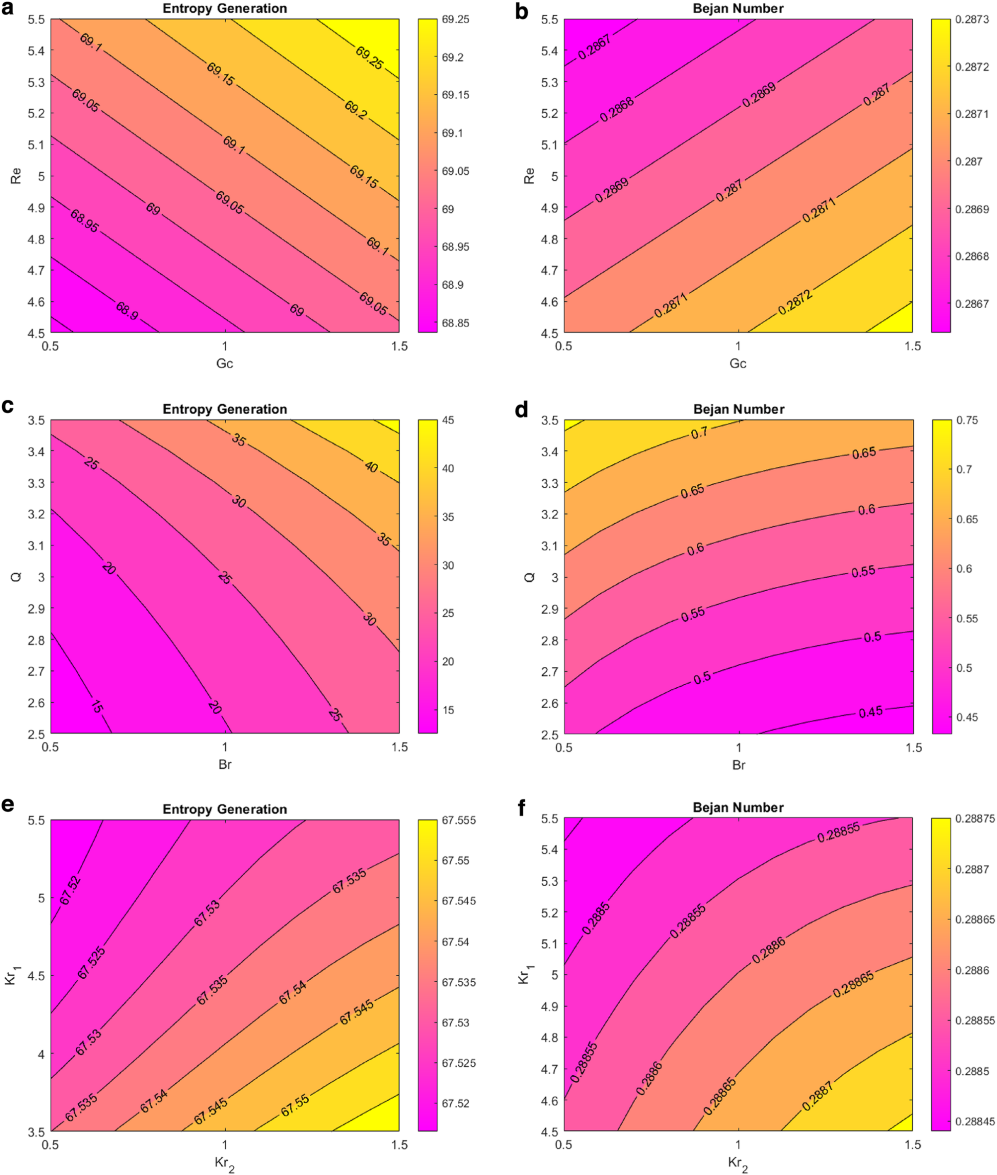

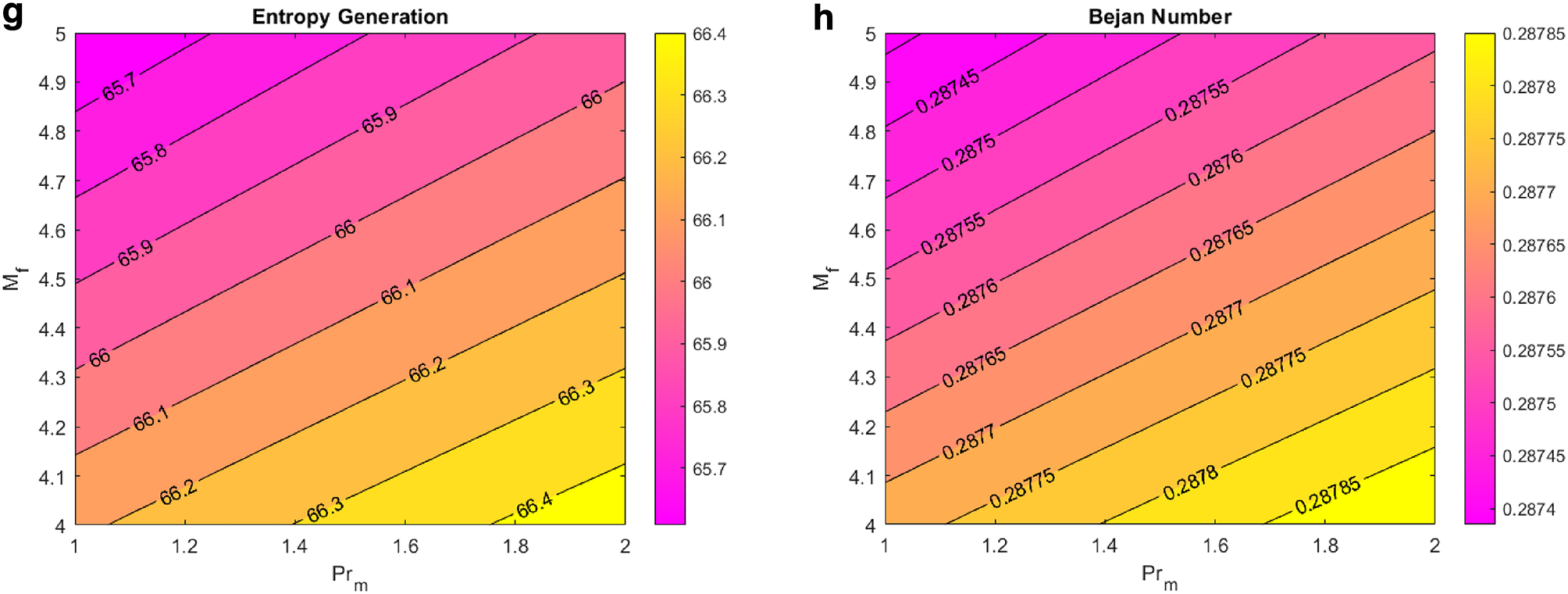
(**a**) EG VS Re VS Gc. (**b**) Be VS Re VS Gc. (**c**) EG VS Q VS Br. (**d**) Be VS Q VS Br. (**e**) EG VS Kr_1_ VS Kr_2_. (**f**) Be VS Kr_1_ VS Kr_2_. (**g**) EG VS M_f_ VS Pr_m_. (**h**) Be VS M_f_ VS Pr_m_*.*

Figure 10 shows the entropy generation and Bejan number contour plots for various flow parameters. Figure 10a,b show the entropy generation and Bejan number plots for the Reynolds and solutal Grashof numbers. The figure shows that the entropy generation increases by enlarging the Reynolds number, and the Bejan number decreases. Increased solutal Grashof number increases both entropy generation and Bejan number. Physically, the viscous forces decrease for enhanced Reynolds and solutal Grashof number values, and the Brownian motion increases. The rapidly moving nanoparticles increase the entropy of fluid for both fluid parameters. However, with the Bejan number being the entropy ratio, the effect could not become dominant for these particular values of the Reynolds number, and the Bejan number decreased. Figure 10c,d signify the importance of the entropy generation, Bejan number for the heat source, and Brinkman number. The entropy generation and Bejan number increase for enhanced heat source values. Physically, larger values of the heat source parameter provide more heat energy to the fluid, eventually increasing the entropy and Bejan number. Enhanced values of the Brinkman number decreased the Bejan number and increased the entropy generation. Physically, viscosity is enhanced by a higher Brinkman number, which increases the likelihood of kinematic collision. As a result, the rate of entropy rises. Furthermore, viscous dissipation’s irreversibility surpasses heat transfer’s irreversibility as the Brinkman number increases. The Brinkman number measures the ratio of the heat provided by the liquid molecules to the heat dissipated (produced) through viscous dispersion. As a result, a rise in the Brinkman number reveals that viscous dispersion produces a sizeable portion of the heat generation compared to molecular conduction. Figure 10e,f signify the entropy generation and Bejan number behavior for chemical reactions and endothermic-exothermic parameters. Increasing the chemical reaction parameter, the entropy and Bejan number decrease. This makes sense, as the collision and sticking between the nanoparticles of the fluid is enhanced due to the expansion of the chemical reaction parameter, decreasing the concentration of nanoparticles, and reducing the heat generation in the fluid. This figure also shows that the entropy and Bejan number elevated for improved values of the endothermic–exothermic reaction parameter. When this parameter takes larger positive values, it signifies the dominating effect of an endothermic reaction. During an endothermic reaction, the nanoparticles absorb energy from the surroundings, enhancing the fluid’s generated entropy. Figure 10g,h depict the effect of induced magnetic fluid number and magnetic Prandtl number on entropy generation and Bejan number. Raised values of the induced magnetic field drop the entropy and Bejan number. The more magnetic forces the nanoparticles, the more the Lorentz forces on them. These forces decrease the nanoparticles’ velocities. For decreased velocity, a lesser entropy is generated. This figure also illustrates that the entropy and Bejan number increase for larger values of magnetic Parndtl number. This can be justified as the magnetic Prandtl number is mathematically proportional to the heat transfer rate.

### Physical significance quantities

The skin friction coefficient is a dimensionless quantity that characterizes the frictional forces exerted by a fluid on a solid surface. It is an important parameter in the study of nanofluids and has important practical applications in various industries, including transportation, electronics, oil recovery, and biomedical applications. The mass transfer rate (Sherwood number) is defined as the rate at which the mass of a particular species is transported across a surface in a given direction due to the concentration gradient of that species. It has significant applications in various engineering fields, such as heat transfer, energy systems, chemical engineering, environmental engineering, and biotechnology. Heat transfer rate in nanofluids refers to the rate at which heat energy is transferred through a nanofluid. The concept is used in electronics cooling applications, solar thermal systems, nuclear reactors, and heat exchangers. Table 3 displays the relationship between skin friction coefficient, heat transfer rate (Nusselt number), and mass transfer rate (Sherwood number) and nanoparticle concentration. When the concentration of aluminium nanoparticles is increased, it is seen that the skin friction coefficient and mass transfer rate rise, however the opposite impact is seen for the rate of heat transfer coefficient. The skin friction coefficient, the mass transfer coefficient, and the heat transfer rate all rise with increased concentrations of copper nanoparticles, but the trend for the latter two reverses. The same effect is seen for iron oxide concentration.

**Table 3:** Physical significance quantities for nanoparticle concentrations.

Flow parameters	Skin friction coefficient	Nusselt number	Sherwood number
${\phi }_{\text{Al}}=0.01$	5.9089	− 2.6403	1.6472
${\upphi }_{\mathrm{Al}}=0.03$	6.0834	− 2.7188	1.7331
${\upphi }_{\mathrm{Al}}=0.05$	6.2259	− 2.9585	1.9728
${\upphi }_{\mathrm{Cu}}=0.01$	5.9089	− 2.6403	1.6472
${\upphi }_{\mathrm{Cu}}=0.03$	6.0813	− 2.6740	1.6889
${\upphi }_{\mathrm{Cu}}=0.05$	6.2232	− 2.8767	1.8921
${\upphi }_{{\mathrm{Fe}}_{2}{\mathrm{O}}_{3}}=0.01$	6.0789	− 2.6403	1.6472
${\upphi }_{{\mathrm{Fe}}_{2}{\mathrm{O}}_{3}}=0.03$	6.2196	− 2.7865	1.7997
${\upphi }_{{\mathrm{Fe}}_{2}{\mathrm{O}}_{3}}=0.05$	6.3356	− 3.0715	2.0844

## Conclusion

This work investigates the ternary hybrid nanofluid made of copper (Cu), aluminium (Al), and iron oxide (Fe_2_O_3_) with blood as the base fluid for the combined effect of viscous dissipation, gravity, external and induced magnetic field, highly porous medium, thermophoresis, Brownian motion, exponential heat source/sink, activation energy, and endothermic-exothermic chemical reactions are investigated. Velocity, temperature, concentration, and induced magnetism profiles, skin friction coefficient, entropy production, Nusselt number, Sherwood number, Bejan number, and mass transfer rates are discussed visually with appropriate arguments. The study's key findings include the following:

- The fluid temperature increases for the enhanced values of heat source, thermophoresis, Brownian motion, and endothermic-exothermic chemical reaction parameter.
- By raising the fitting constant, concentration ratio, chemical reaction parameter, and activation energy parameter, the concentration rises.
- The induced magnetism goes up on boosting the Reynolds, magnetic Prandtl, Darcy, and thermal Grashof numbers.
- The fluid velocity increases by decreasing the Forchheimer number. It first increased and then decreased for enhanced solutal Grashof number, channel inclination, and induced magnetic field number.
- The entropy generation improves for increasing values of Reynolds, solutal Grashof, heat source/sink, Brinkman, magnetic Prandtl, endothermic–exothermic reaction parameter while reverse effect is noticed for chemical reaction and induced magnetic field parameter.
- Bejan number increases by reducing the Reynolds number, Brinkman number, chemical reaction parameter, induced magnetic field parameter and enhancing the solutal Grashof, heat sink/source parameter, magnetic Prandtl number, endothermic–exothermic reaction parameter.
- Skin friction coefficient, the Nusselt number, and the Sherwood number rise as the concentrations of Al, Cu, and Fe_2_O_3_ rise.

The study finds applications in enhancing the design and efficiency of biomedical devices and drug delivery systems, optimize heat transfer processes in microfluidic systems, improve the performance of magnetohydrodynamic power generators, optimize energy conversion in renewable energy systems, and develop efficient cooling systems for electronic devices. Additionally, the findings can also be relevant in the development of advanced materials with tailored thermal properties, optimization of microscale chemical reactions, and understanding biological transport phenomena in living organisms. The research opens possibilities for numerous applications in the areas of biomedical engineering, energy systems, microfluidics, and materials science.

Validating the results of present study would require conducting experiments that closely mimic the conditions and parameters studied in the theoretical analysis. Experimental setups could involve the use of specially designed channels or flow systems that incorporate ciliated walls, magnetic field induction, and the ternary hybrid Jeffery nanofluid. Measurements of fluid velocity, temperature, and concentration gradients, as well as entropy generation rates, would need to be taken and compared with the predicted values from the theoretical model. These experiments would provide valuable empirical evidence to support the validity and applicability of the theoretical findings.

Further research avenues include exploring more biologically accurate models of cilia behavior, investigating multi-scale variations in nanoparticle distribution, employing advanced numerical techniques for enhanced accuracy, conducting experimental validation in controlled medical scenarios, and collaborating across disciplines to develop practical medical applications, such as optimized drug delivery and artificial organ design.

## Limitations

The study considers some limitations and assumptions:

1. The investigation assumes synthetic cilia, thereby simplifying the behavior of real biological cilia. This simplification may overlook the intricate dynamics and interactions that natural cilia exhibit within physiological contexts.
2. The analysis focuses on a specific ternary hybrid nanofluid (Al–Cu–Fe2O3/Blood) with uniform nanoparticle distribution. Consequently, the study neglects potential variations in nanoparticle concentration that might exist in real-world applications.
3. The utilization of the long-wavelength and low Reynolds number approximations could introduce limitations in predicting accurate outcomes, particularly when dealing with scenarios characterized by higher flow rates or substantial variations.
4. Exploration confines itself to a single channel geometry, restricting the generalizability of findings to more complex and diverse anatomical structures.

## Data Availability

All data generated or analysed during this study are included in this article.

## Subscripts

${{thnf}}$: Ternary hybrid nanofluid
$${{hnf}}$$: Hybrid nanofluid
$${{nf}}$$: Nanofluid
$${{f}}$$: Fluid

## Symbols

$\left( {{{X}}^{{*}} ,{{Y}}^{{*}} } \right)$: Fixed frame (in cartesian coordinates)
$\left( {{{x}},{{y}}} \right)$: Wave frame (in cartesian coordinates)
$\left( {{{U}}^{{*}} ,{{W}}^{{*}} } \right)$: Velocity of HNF in $\left( {X^{*} ,Y^{*} } \right)$ direction
$\left( {{{u}},{{w}}} \right)$: Velocity of HNF in $\left( {x,y} \right)$ direction
$${{t}}$$: Time
$${{p}}$$: Pressure
$${{T}}$$: Temperature
$${{C}}$$: Concentration
${{M}}$: Induced magnetic field
$${{S}}$$: Stress tensor
$${{v}}$$: Kinematic viscosity
$${{\mu}}$$: Dynamic viscosity
$${{c}}_{{{p}}}$$: Specific heat
$${{\sigma}}$$: Electrical conductivity
$${{k}}$$: Thermal conductivity
$${{\rho}}$$: Density
${{\eta}}$: Symmetric channel inclination to horizontal
$${{g}}$$: Gravitational acceleration
${{B}}_{0}$: Uniform magnetic field strength
$\left( {{{T}}_{0} ,{{T}}_{1} } \right)$: Temperature at walls
$\left( {{{C}}_{0} ,{{C}}_{1} } \right)$: Concentration at walls
$\left( {{{m}}_{{{x}}} ,{{m}}_{{{y}}} } \right)$: Induced magnetic field components in $\left( {x,y} \right)$ directions
${{\beta}}_{{{t}}}$: Volumetric thermal coefficient
${{\beta}}_{{{c}}}$: Volumetric concentration coefficient
${{\rho}}_{{{p}}}$: Density of nanoparticles
$${{\mu}}_{{{m}}}$$: Magnetic permeability
${{\tau}}$: Effective heat capacity
${{k}}_{1}$: Permeability of porous medium
${{F}}_{0}$: Porous medium coefficient
$${{k}}_{{{b}}}$$: Boltzmann constant
${{D}}_{{{B}}}$: Brownian motion coefficient
${{D}}_{{{T}}}$: Thermophoretic diffusion coefficient
${{m}}_{0}$: Constant of magnetic field
$${{\alpha}}_{{{m}}}$$: Magnetic diffusivity
${{q}}_{0}$: Heat source/sink
${\varsigma }$: Endothermic–exothermic reaction coefficient
$${{E}}_{{{a}}}$$: Activation energy
${{k}}_{{{r}}}$: Chemical reaction constant
$${{n}}$$: Fitting constant
${{E}}_{{{x}}}$: Electrokinetic body force
$${{\lambda}}_{2}$$: Retardation time
${{d}}$: Channel half width
$${{\lambda}}$$: Wavelength
$${{a}}_{{{i}}} \left( {{s}} \right)$$: Wave amplitudes
$${{c}}$$: Wave speed
$${{\delta}}$$: Wavenumber
${{\sigma}}^{{*}}$: Stefan–Boltzmann constant
$${{k}}^{{*}}$$: Absorption coefficient
$$\dot{{\gamma }}$$: Shear rate
$+ {{H}}$: Upper cilia wall
$- {{H}}$: Lower cilia wall
${{G}}$: Non-uniform parameter
${{L}}$: Cilia length parameter
${{b}}_{{{i}}} \left( {{s}} \right)$: Physical parameters of geometry
${{\lambda}}_{1}$: Ratio of relaxation to retardation times
$${{\psi}}$$: Stream function
${\varphi }$: Magnetic stream function
${{F}}$: Time-average flow rate
